# Probiotics—role in alleviating the impact of alcohol liver disease and alcohol deaddiction: a systematic review

**DOI:** 10.3389/fnut.2024.1372755

**Published:** 2024-08-30

**Authors:** Grisilda Vidya Bernhardt, Pooja Shivappa, Janita R. Pinto, Rashmi KS, Jayachithra Ramakrishna Pillai, Suresh Kumar Srinivasamurthy, Vijay Paul Samuel

**Affiliations:** ^1^Department of Biochemistry, RAKCOMS, Ras Al-Khaimah Medical and Health Sciences University, Ras Al-Khaimah, United Arab Emirates; ^2^Department of Biomedical Sciences, Gulf Medical University, Ajman, United Arab Emirates; ^3^Department of Physiology, Kasturba Medical College Mangalore, Manipal Academy of Higher Education, Manipal, Karnataka, India; ^4^Department of Pharmaceutical Chemistry, RAKCOPS, Ras Al-Khaimah Medical and Health Sciences University, Ras Al-Khaimah, United Arab Emirates; ^5^Department of Pharmacology, RAKCOMS, Ras Al-Khaimah Medical and Health Sciences University, Ras Al-Khaimah, United Arab Emirates; ^6^Department of Anatomy, RAKCOMS, Ras Al-Khaimah Medical and Health Sciences University, Ras Al-Khaimah, United Arab Emirates

**Keywords:** probiotics, alcohol addiction, alcohol use disorder, alcohol liver disease, gut microbiota, dysbiosis, neuroinflammation, gut-brain axis

## Abstract

**Background:**

There are few efficient treatment options for alcohol addiction, which continues to be a serious public health concern. The possible contribution of gut microbiota to the onset and progression of alcohol addiction has been brought to light by recent studies. Probiotics have become a cutting-edge intervention in the treatment of alcohol consumption disorder because of its favorable effects on gut health. The purpose of this systematic review is to assess the body of research on the advantages of probiotics in treating alcoholism and associated neuroinflammatory conditions.

**Methods:**

To find pertinent research published from January 2012 to 2023, a thorough search of electronic databases, including PubMed, Scopus, Google Scholar and Web of Science, was carried out. Included were studies looking at how probiotics affect neuroinflammation, gut- brain axis regulation, alcohol addiction, and related behaviors.

**Findings:**

Several investigations have shown how beneficial probiotics are in reducing systemic inflammation and alcoholic liver disease (ALD). Probiotic treatments successfully corrected the imbalance of microbiota, decreased intestinal permeability, and stopped the passage of bacterial constituents such lipopolysaccharides (LPS) into the bloodstream. Additionally, probiotics helped to regulate neurotransmitter pathways, especially those connected to GABA, glutamate, and dopamine, which are intimately linked to behaviors related to addiction. Furthermore, it was shown that probiotics altered the expression of neurotransmitter signaling and dopamine receptors.

**Conclusion:**

There is strong evidence from this systematic study that probiotics have potential advantages in treating alcohol addiction. The potential of probiotic therapies is demonstrated by the way they modulate important neurotransmitter pathways implicated in addiction, decrease neuroinflammation, and restore the balance of gut flora. To fully investigate the therapeutic potential of probiotics in treating alcohol addiction and enhancing the general wellbeing of those afflicted by this condition, more research is necessary.

## Introduction

Alcohol use disorder (AUD), often known as alcohol addiction, is a prevalent issue affecting a significant portion of the adult population, with around 4% of adults being impacted by it (1). According to a study conducted in 2016, 2.2% of deaths in females and 6.8% of deaths in males were found to be related to alcohol consumption. The consumption of alcohol was found to be responsible for 2.2% of deaths among females and 6.8% of deaths among males. Furthermore, it was noted that 2.3% of female and 8.9% of male disability-adjusted life years were linked to alcohol-related causes (2). Alcohol consumption has been found to have a considerable negative impact on both the duration and quality of life for individuals diagnosed with AUD as well as their relatives (3). Alcohol addiction is characterized as a neurological disorder that damages the brain’s reward pathway. People with AUD are more likely to experience co-occurring conditions such depression, anxiety, cognitive decline, and use of illegal drugs. The co-occurrence of alcoholism with liver diseases, such as liver cirrhosis and alcoholic hepatitis, is extremely common and has substantial worldwide consequences for mortality and morbidity (4). Alcohol addiction is a persistent medical condition characterized by periods of relapse and remission (5).

As per the Diagnostic and Statistical Manual of Mental Disorders 5th edition (DSM-V), AUD is characterized by a constellation of cognitive impairment and unregulated conduct, encompassing the development of tolerance, withdrawal symptoms, escalating consumption, and a strong need for alcohol (6). Alcohol addiction, similar to other forms of substance addiction, induces the pursuit of alcohol and the perpetuation of alcohol consumption, impacting variety of neurotransmitter systems, such as glutamate, opioid peptides, dopamine, serotonin, and glutamate γ-aminobutyric acid (GABA) (7). In both Europe and the United States, medical interventions for alcohol addiction involve the utilization of various treatments that specifically target the aforementioned neurotransmitter systems. These treatments include naltrexone, an antagonist of opioid receptors, nalmefene, a modulator of opioid receptors, acamprosate, which affects multiple targets and also disulfiram, which acts as an inhibitor of aldehyde dehydrogenase. Additionally, psychological interventions like motivational interviewing and cognitive behavior therapy are also employed in the treatment of alcohol addiction (8–10).

The chronic consumption of alcohol is widely recognized as a significant risk factor for liver injury (11). Among individuals diagnosed with AUD, alcoholic liver disease (ALD) is considered a prominent source of morbidity. Alcohol consumption is known to cause several clinical manifestations of liver damage, including steatosis, ASH, alcoholic hepatitis, fibrosis, and cirrhosis. These conditions are widely recognized as significant public health concerns, as stated by the World Health Organization in 2018 (2,3,12). On a global scale, the consumption of alcohol has a notable socioeconomic influence on the population, as seen by a heightened death rate resulting from alcohol cirrhosis, which is directly linked to escalated levels of alcohol intake. The projected trajectory suggests that there will be a rise in both alcohol intake and the prevalence of ALD in the forthcoming decades. This trend is closely intertwined with the psychosocial challenges prevalent in today’s culture (9, 12). As a result, healthcare systems are faced with a substantial and growing need for treatment of ALD. Currently, abstinence-based therapies continue to be the fundamental approach in the clinical therapy of ALD. Nevertheless, because of the notable recurrence rate reported in individuals with AUD, there is a growing demand for the development and implementation of novel therapeutic interventions for this condition (12).

A substantial amount of research has been conducted in recent years to examine the role of the microbiota-gut-liver axis in the pathophysiology of ALD. Probiotic-based interventions have been tested in patients with ALD, with positive results in their treatment. Other techniques aiming at restoring the homeostatic function of this axis have also been tested.

The positive effects of probiotics have been extensively investigated in numerous pathologies, including gastrointestinal ailments, as well as in the treatment of various central nervous system (CNS) disorders (13). Studies have focused on the restoration of bacterial balance and the potential to influence systemic and CNS inflammation. Additionally, the possible advantages of probiotics in relation to CNS and mental health have led to a suggestion to classify them as “psychobiotics.” (13, 14). This classification is based on their potential to exhibit anti-inflammatory, antidepressant, and anti-anxiety qualities with minimal adverse effects. Utilization of probiotics may have potential benefits in enhancing cognitive performance in individuals with Alzheimer’s disease and Autism spectrum disorders, as well as in reducing the development of tolerance to the analgesic effects of morphine (14).

This systematic review aims to study the impact of probiotic supplementation on alcohol addiction including its effects on alcohol intake, cravings, guts microbiota composition, and related behavioral and physiological impacts.

## Methods

The systematic review was undertaken in accordance with the PRISMA criteria (15) to ensure the reliability and comprehensiveness of the data and findings. The English databases searched included PubMed, Embase, the Cochrane Library, and the Web of Science and Scopus. The language used was English, according to the authors’ level of comprehension.

### Literature search strategy

An electronic search was conducted in the databases with free terms, subject heading terms and key words. The keywords are as follows, (“Probiotic” OR “Gut microbiota” OR “Microbiome”) AND (“Alcohol addiction” OR “Alcohol use disorder” OR “Alcoholism”) AND (“Probiotic intervention” OR “Probiotic therapy”) AND (“Alcohol dependence” OR “Alcohol craving”) AND (“Review article” OR “Clinical trials”) AND (“Efficacy of probiotics” OR “Probiotic treatment for alcohol addiction”) AND (“Substance use disorder” OR “Alcohol withdrawal”) AND (“Evidence synthesis” OR “Patient outcomes”). Studies that provide data on the effects of probiotics in the context of alcohol addiction published from January 2012 to November 2023 were included in this review. This period was selected to ensure the inclusion of the most up-to-date and relevant studies, reflecting the latest advancements and current trends in this rapidly evolving field. Preliminary searches indicated that older studies did not significantly contribute new knowledge or insights relevant to the objectives of this study. Table 1 presents the specific inclusion and exclusion criteria based on the PICOS (i.e., population, interventions, comparisons, outcomes, and study design) strategy.

**Table 1:** PICOS criteria of inclusion and exclusion.

Criteria	Inclusion	Exclusion
Population	Human participants with a history of alcohol addiction or alcohol use disorder. Animal models of ALD treated with probiotics as therapeutic agents against liver damage.	Populations with different substance use disorders not related to alcohol.
Intervention	Use of probiotics as an intervention for individuals with ALD and alcohol addiction. Modulation of gut microbiota through probiotic administration.	Studies that do not use probiotics as an intervention.
Comparisons	Control group (placebo or no treatment) to compare the effects of probiotics.	No specific exclusion criteria for comparisons, but implied exclusion if control groups are not used.
Outcomes	Quantitative data on outcomes such as alcohol consumption reduction, craving reduction, changes in gut microbiota, or other physiological/psychological measures.	- Studies that do not report relevant quantitative outcomes related to alcohol addiction, probiotics, or relevant measures.
Study design	Randomized controlled trials, clinical trials, or observational studies (cohort, case–control).	Letters, editorials, commentaries, and conference abstracts that do not provide sufficient original data.

### Study selection

**Figure 1:**
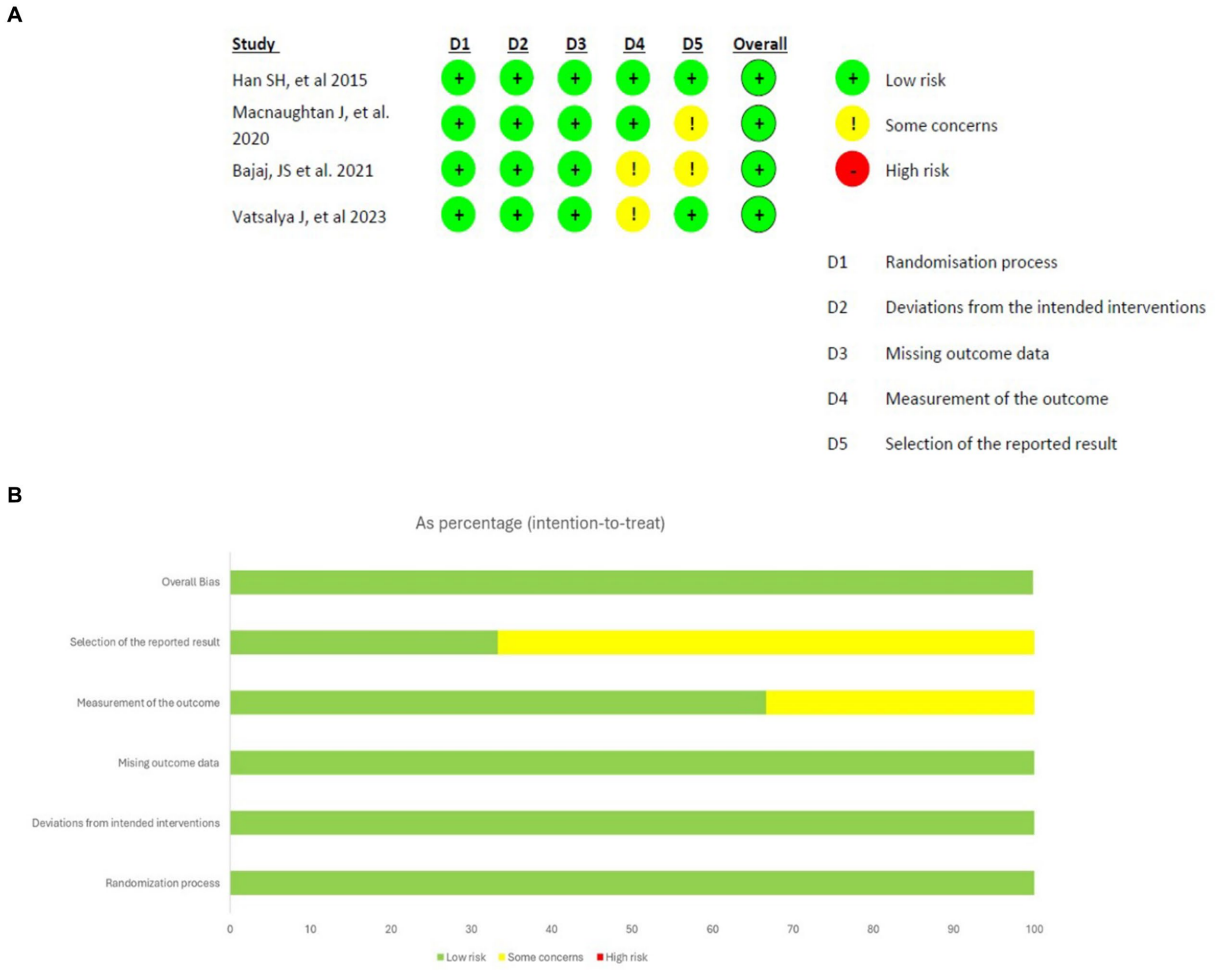
Results of risk of bias analysis of intention-to-treat studies. **(A)** Risk of Bias by article included on each domain. **(B)** Overall risk of Bias percentage on each domain (16).

Prior to conducting the database search, all researchers underwent a comprehensive training session to ensure a uniformity of ideas and a thorough understanding of the inclusion and exclusion criteria. The databases were thoroughly searched by the researchers, who then independently assessed the studies. The initial screening of the studies involved evaluating their titles and abstracts, followed by a thorough examination of the complete texts of the selected studies to determine their eligibility based on the predefined inclusion and exclusion criteria. Ultimately, the researcher conducted a comparison of the screened full-texts. Divergence and discrepancies were effectively addressed by a process of dialogue and vote among the researchers. The data extraction process involved gathering information from eligible studies using a predetermined template. This template encompassed several aspects such as the study’s title, authors, publication year, research design, participant characteristics, intervention details, outcome measures, results and statistical findings, as well as the conclusions and implications drawn from the study. The assessment of the methodological quality of the studies (Figure 1) included in the analysis was conducted using suitable techniques, such as the Cochrane Risk of Bias tool for clinical trials (16).

## Results

**Figure 2:**
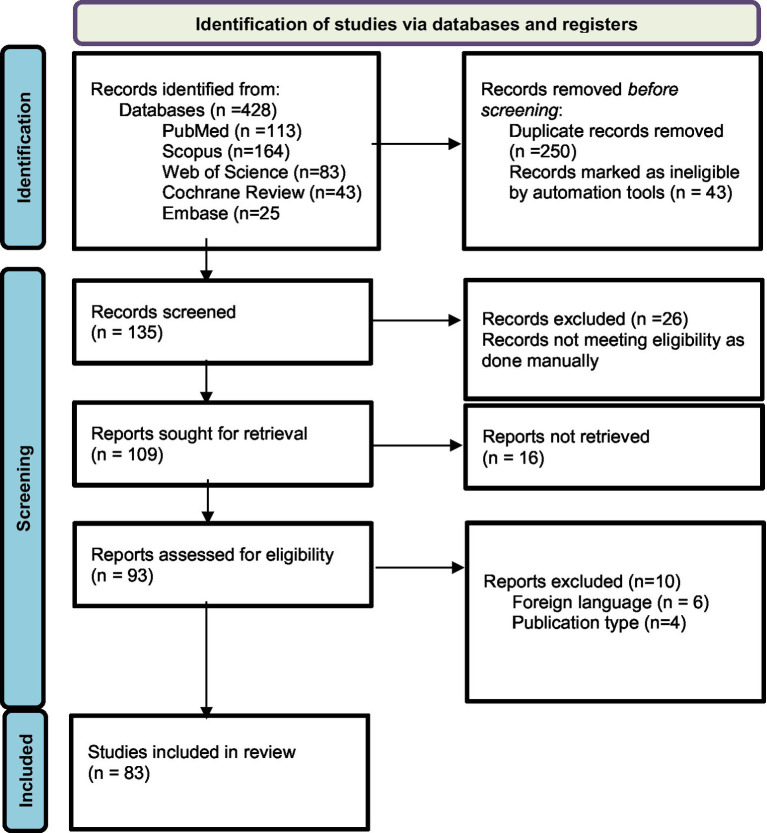
Flowchart of PRISMA.

The original search conducted on databases resulted in a total of 428 articles and abstracts. Among these, 135 publications were selected based on their relevance to the objectives of the current study after removing duplicate records (*n* = 250) and excluding those marked as ineligible by automation tools (*n* = 43). The automation tool EndNote was configured to exclude records based on predefined criteria, including studies not focusing on probiotics as an intervention. Additionally, non-original data sources such as letters, editorials, commentaries, and conference abstracts were excluded. EndNote efficiently filtered out irrelevant studies, ensuring that only potentially eligible studies were considered for full-text review. After further examination, 26 reports were excluded manually following a full-text review, which revealed they were unrelated to the objectives of our study, such as studies focused on unrelated substance use disorders. Out of 109 reports sought for retrieval, 93 were found to be relevant and retrievable. Further exclusions were made based on foreign language (*n* = 6) and publication type (*n* = 4). This process left 83 articles to be included for review, of which only 4 were randomized controlled trials RCTs involving probiotics and AUD. This selection followed the Preferred Reporting Items for Systematic Reviews and Meta-Analyses (PRISMA) 2020 guideline, as indicated in Figure 2.

### Findings of the study

#### Alcoholic liver disease and therapeutic potential of probiotics

Probiotics are live bacteria that, when consumed in adequate amounts, offer health benefits by supporting the gut microbiota. Alternately, non-digestible fibers called prebiotics are present in foods like whole grains, fruits, and vegetables. Prebiotics support the development and activity of probiotics by providing the necessary fuel for their growth and maintenance in the gut (17).

Excessive alcohol consumption has been shown to negatively impact gut microbiota, leading to dysbiosis characterized by changes in the functions and composition of the gut microbiome (9). Prolonged alcohol consumption is reported to cause intestinal dysbiosis by increasing microbes such as *Corynebacterium* and *Alcaligenes* and a decrease in beneficial bacteria such as *Bacteroidetes, Lactobacillus, Firmicutes*, and *Akkermansia muciniphila*. Numerous studies have demonstrated that probiotics have several benefits on ALD (18, 19). Upon ingestion, 90% of alcohol is absorbed through the small intestine and then distributed throughout the body. However, the liver is central to alcohol metabolism through oxidative and non-oxidative mechanisms, leveraging its rich supply of metabolizing enzymes (9).

Alcohol is primarily metabolized with the help of the enzyme’s alcohol dehydrogenase and acetaldehyde dehydrogenase. It can also be metabolized by the microsomal enzyme system Cytochrome P450 2E1 (CYP2E1), which simultaneously generates reactive oxygen species (ROS). ROS produced during ethanol metabolism increases the demand for antioxidant enzymes like superoxide dismutase (SOD), catalase, and peroxidases, thereby relatively decreasing their levels. Increased ROS production during excessive alcohol metabolism, and decreased antioxidant levels together trigger oxidative stress, which can damage the hepatocytes, leading to ALD (18, 20, 21).

ROS lead to lipid peroxidation, forming malondialdehyde (MDA) and 4-hydroxy-2-nonenal compounds, which then bind with proteins to create damaging adducts that further contribute to inflammation (22). The effects of ROS may also be mediated by activating transcription factors, namely the nuclear factor erythroid 2-related factor 2 (Nrf2) and nuclear factor kappa-light-chain-enhancer of activated B cells (NF-κB), which releases cytokines that are proinflammatory, including interleukin-6 (IL-6) and tumor necrosis factor (TNF), and hastens the development of liver diseases (22–24). Additionally, mitochondrial and peroxisomal enzymes involved in β-oxidation are compromised by ROS, leading to fatty acid accumulation in the liver and promoting hepatic steatosis and ALD (9). Concurrently, oxidative stress adversely affects liver mitochondria, altering membrane permeability and energy production, leading to cell death and the subsequent development of hepatic fibrosis and cirrhosis due to stellate cell activation and extracellular matrix remodeling (9, 11). Moreover, oxidative stress impairs the regenerative capacity of hepatocytes, further complicating liver disease progression (11). Alcohol consumption significantly impacts lipid metabolism in the liver by altering the expression of key proteins such as peroxisome proliferator-activated receptor alpha (PPAR-α) and microsomal triglyceride transfer protein (MTP) (25, 26). PPAR-α, a nuclear receptor crucial for enhancing fatty acid oxidation, experiences reduced expression under the influence of alcohol, leading to compromised lipid metabolism and triglyceride accumulation, thereby contributing to ALD and hepatic steatosis (27). Additionally, MTP plays a vital role in assembling and secreting very low-density lipoproteins (VLDL) for efficient lipid transport; alcohol-induced downregulation of MTP limits triglyceride export from the liver, exacerbating the risk of fatty liver disease and underscores the detrimental impact of alcohol on liver health (28). Substantial quantities of acetaldehyde produced by alcohol dehydrogenase also play a crucial role in alcohol-induced liver damage (3, 8).

While the liver is the primary site of alcohol metabolism, the consumption of alcohol leads to dysregulation of intestinal bacteria and damage to the intestinal mucosa (29). Alcohol and its metabolites increase intestinal permeability and disrupt barrier functions through microtubule damage by activating nuclear transcription factors like nuclear factor kappa B (NF-κB) and inducible nitric oxide synthase, leading to inflammation (18, 30). Additionally, intestinal flora displaced by excessive acetaldehyde disrupts the mucosal barrier of the intestine (18). The altered gut microbiota can also metabolize ethanol, further contributing to the production of acetaldehyde in the gut (8). This situation is exacerbated by the compromised ability of the liver to metabolize acetaldehyde efficiently due to alcohol-induced liver damage. The increased intestinal permeability allows more acetaldehyde to enter the bloodstream and reach the liver (9, 11). Dysbiosis can lead to the overproduction of ROS as certain harmful bacteria, when overrepresented in the gut microbiome, induce inflammation and increase ROS production (31). During dysbiosis, the increase in gram-negative bacteria disrupts bile acid balance, affects tight and adherent junctions, and compromises the intestinal barrier function. This enhances intestinal permeability, allowing bacterial products and endotoxins such as lipopolysaccharides (LPS) and pathogen-associated molecular patterns (PAMPs) to enter the systemic circulation and reach the liver via the portal vein, inducing inflammation in hepatic and other tissues (32). Endotoxins produced by gut bacteria bind to pattern recognition receptors such as Toll-like receptor 4 (TLR4) and its co-receptors, cluster of differentiation 14 (CD14), and myeloid differentiation factor 2 (MD2), expressed by Kupffer cells, macrophages, and other liver cell types (18). These endotoxins and PAMPs activate liver macrophages and Kupffer cells, increasing the production cytokines that are pro-inflammatory like TNF-α, IL-6, and interleukin-1 beta (IL-1β). Increased ROS production reduces phagocytosis and impairs immune regulation (33). These processes facilitate the leakage of endotoxins into the bloodstream, leading to the proliferation of hepatic stellate cells, inflammation, immune system alterations, damage to hepatocytes, and changes in metabolic pathways within the liver (11).

Proper activation of the aryl hydrocarbon receptor (AhR) by its natural ligands, such as microbial-derived tryptophan metabolites, is important for maintaining intestinal homeostasis and preventing dysbiosis (34). Dysbiosis decreases AhR activity, impairs AhR signaling, and promotes inflammation in the gut and adds to the increased intestinal permeability and LPS translocation. The AhR signaling pathway, when dysregulated, can contribute to ALD (34, 35). The inflammatory cytokines produced due to LPS, such as TNF-α and IL-1β, can also activate via the mitogen-activated protein kinases (MAPK) pathway. The activated NF-κB translocates into the nucleus and initiates the transcription of a variety of genes involved in the inflammatory response, including cytokines, chemokines, and adhesion molecules (36). This increased cytokine production perpetuates the inflammatory response, creating a cytokine-NF-κB loop that triggers a cascade of inflammatory reactions within cells. Alcohol consumption exacerbates this situation by further increasing oxidative stress and inflammation (9, 11, 17). Critical factors in the progression of ALD through intestinal dysbiosis include intestinal barrier dysfunction, altered fatty acid metabolism, activation of TLR4, inflammation, compromised immunity, translocation of toxic substances from pathogenic bacteria, and changes in AhR signaling pathways (34–36).

Probiotics can decrease systemic inflammation by reducing proinflammatory markers such as TNF-α and IL-6 through downregulating the expression of NF-κB. They modulate the immune response to promote an anti-inflammatory environment (36), including upregulating anti-inflammatory cytokines like interleukin-10 (IL-10). Additionally, probiotics enhance intestinal cell integrity, decrease permeability (37, 38), regulate gut flora, and reduce hepatic endotoxin levels, leading to a decrease in proinflammatory markers. Thus, probiotics beneficially impact ALD (39, 40). Probiotics can regulate many pathophysiological pathways associated with the progression of liver damage. The mechanisms by which probiotics deliver their benefits include reducing dysbiosis, mitigating endotoxemia, increasing the adhesion of specific probiotic bacteria to intestinal cells, and enhancing intestinal epithelial integrity (41). Probiotics inhibit the translocation of bacterial metabolites to the liver, leading to a reduction in the inflammatory response in the liver (18). They also have antioxidant effects and influence liver lipid metabolism. Additionally, probiotics restore microbiota balance, create an anti-inflammatory environment, regulate the immune system, alter TLR expression, suppress intestinal inflammation, modulate cytokine production, and produce antimicrobial substances (18, 19, 42). Since ALD significantly impacts gut microbiota, modulation of the microbiome may be a potential therapeutic approach for ALD (43, 44).

The hepatotoxic effects of alcohol are mediated by its interference with lipid metabolism, disruption of the mucosal barrier, augmentation of the inflammatory reaction, and promotion of oxidative stress, which can be mitigated by probiotic therapy along with substantial alterations in liver-specific biological enzymes, aspartate transaminase (AST), alanine transaminase (ALT), and gamma glutamate transferase (9). Alcohol-induced dysfunction of the mitochondria through CYP2E1 enzyme activation and the subsequent substantial release of enzyme AST from the matrix of the mitochondrial were also alleviated by probiotics (42). *Lactococcus lactis, Bifidobacterium bifidum, Bifidobacterium animalis* subsp. *lactis* have demonstrated significant antioxidant activity both *in vitro* and *in vivo*, alleviating oxidative stress by mediating lipid peroxidation, glutathione (GSH) levels, and enhancing the activity of antioxidant enzymes like catalase, SOD, and glutathione peroxidase (GPX). Additionally, strains like *Bifidobacterium animalis, Lactobacillus rhamnosus*, and *Bacillus* LBP32 have also been found to exhibit potent antioxidant capacity and alleviate oxidative damages in various studies (45). These probiotics’ antioxidant mechanisms involve down-regulating the activities of enzymes involved in the production of ROS, secreting antioxidant metabolites, and modulating antioxidant activities (46).

Probiotics can influence the production of AhR ligands in the gut. By modulating the composition of the gut microbiota, probiotics can enhance the production of compounds that serve as AhR ligands. For example, certain *Lactobacillus* strains can metabolize dietary tryptophan into indole derivatives that activate AhR, thus potentially contributing to gut homeostasis and immune regulation (47). Probiotics, particularly strains of *Lactobacillus* and *Bifidobacterium,* exhibit beneficial properties by enhancing antioxidant defenses through various mechanisms. These probiotic microorganisms scavenge ROS and reactive nitrogen species, inhibit pro-oxidative enzymes, and stimulate the production of antioxidant enzymes (48–50). Additionally, *Lactobacillus* species produce functional compounds like exopolysaccharides that aid in reducing oxidative damage and activating transcription factors within the host, crucial for managing cellular responses to oxidative stress (51).

*Lactobacillus rhamnosus* (LGG) has the potential to effectively repair pre-existing alcoholic hepatic steatosis and associated damage. The effect of probiotics on liver function **can** be observed through their ability to restore levels of ALT, AST, total bilirubin and lactate dehydrogenase, the recognized indicators of liver injury (18). Utilization of *Lactobacillus* species; such as *Lactobacillus plantarum* and fructo-oligosaccharides, leads to a decrease in the production of primed TNF-α by peripheral blood mononuclear cells in individuals with cirrhosis (52). It has been shown in *in vitro* studies that Bifidobacteria have the ability to stimulate the synthesis of IL-10 by human dendritic cells cultivated in a laboratory setting. This cytokine has the capacity to regulate the immune system (38). *Bifidobacterium* and *Lactobacillus* can lower ROS and decrease cytokine levels by decreasing TLR-mediated endotoxins and MDA by suppressing the inflammatory response and mitigating the oxidative impact of alcohol (53, 54). Conversely, these probiotics increase the levels of antioxidants such as SOD and GSH, further helping to decrease oxidative stress. The antioxidant capabilities of probiotics, especially Lactobacillus and Bifidobacterium strains, make them promising adjunct treatments for conditions characterized by oxidative stress, emphasizing their potential in promoting overall health and well-being (49, 50).

**Figure 3:**
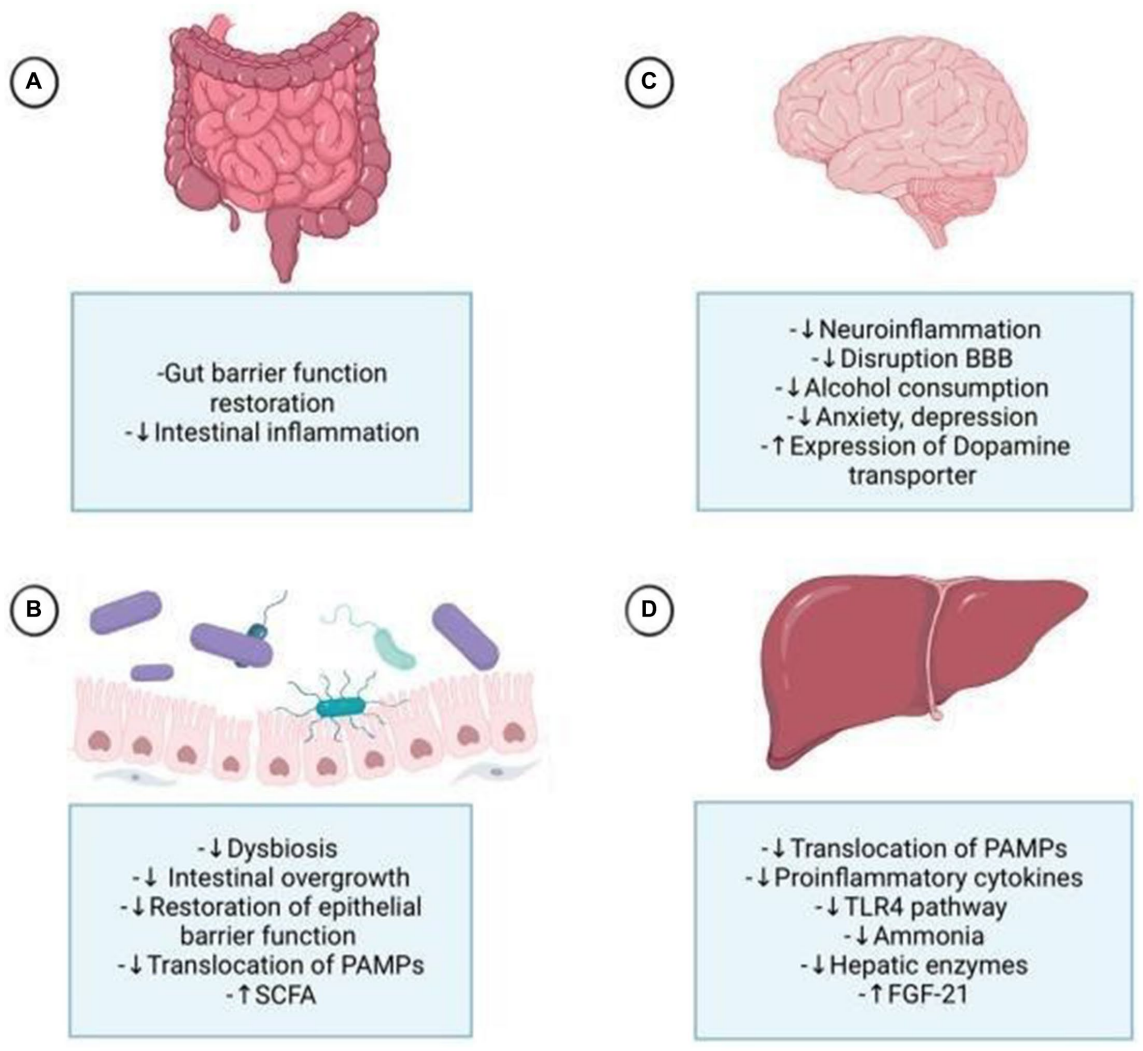
Probiotics affect the alcohol-related gut-microbiota-liver-brain axis, impacting each organ through direct and indirect mechanisms. **(A)** They enhance digestion, strengthen tight junctions, and protect the intestinal crypts and mucous layer. **(B)** Probiotics mitigate alcohol-induced dysbiosis by reducing harmful bacteria, improving gut permeability, and lowering the translocation of pathogen-associated molecular patterns (PAMPs) to the liver. **(C)** They reduce neuroinflammation by decreasing proinflammatory cytokines, potentially alleviating alcohol-induced anxiety and depression. By controlling gut permeability and substance translocation, probiotics help prevent disruption of the blood-brain barrier (BBB) and neuroinflammation. Probiotics also influence dopamine regulation by increasing dopamine transporter expression. **(D)** Probiotics improve liver health by reducing steatosis, hepatic encephalopathy, and cirrhosis, primarily through the normalization of inflammatory processes by decreasing PAMPs, particularly lipopolysaccharides (LPS), via the Toll-like receptor 4 (TLR4) pathway, and by increasing fibroblast growth factor 21 (FGF21) production. FGF21 impacts the brain by reducing dopamine availability at postsynaptic receptors through increased dopamine transporter transcription in the nucleus accumbens.

Table 2 provides a summarized overview of the various aspects of the Positive effects of probiotics on AUD. Figure 3 represents the effects of probiotics on the alcohol-related gut-microbiota-liver-brain (ALD) axis, illustrating how probiotics influence this axis by impacting the gut, liver, and brain, both directly and indirectly, through their interactions across these organs.

**Table 2:** Clinical benefits of probiotics in AUD -human trials.

Authors	Clinical benefits of probiotics in AUD	Study type
Vatsalya et al., 2023	The effects of LGG therapy on alcohol consumption and liver function in individuals with mild alcohol-related hepatitis evaluated at baseline, 1, 3, and 6 months. A 6-month LGG treatment significantly decreased heavy drinking levels in patients with moderate/mild alcohol-induced hepatitis Significant reduction in biomarkers of liver injury progression and severity, indicating potential therapeutic benefits for liver health Significant improvement in liver function tests (reduction in ALT, AST, and bilirubin levels) in the probiotic group. Positive changes in composition of gut microbiota, with an increase in beneficial bacteria such as *Bifidobacterium* species and the *Lactobacillus*. Significant decrease in inflammatory markers (CRP, TNF-α) in the probiotic group.	Randomized controlled trial involving patients with moderate alcohol-associated hepatitis
Macnaughtan J, et al., 2020	Adults with clinically stable cirrhosis received a daily dose of *Lactobacillus casei* Shirota (6.5 × 10^9^ CFU)/bottle or placebo 3 times per day for 6 months, with follow-up assessments at baseline, mid-intervention, and post-intervention. The cytokine profile was enhanced by probiotic supplementation toward an anti-inflammatory phenotype; this effect seems not dependent to bacterial translocation.	Placebo-controlled Double-blind, randomized trial involving Adults with clinically stable cirrhosis
Bajaj et al., 2021	To study the potential of FMT as a therapeutic intervention for individuals with alcohol use disorder, Patients who received FMT showed a significant reduction in alcohol craving and consumption compared to the placebo group at 15 days post-treatment. The FMT group showed improvements in cognition, psychosocial quality of life, and reductions in inflammatory markers (serum IL-6 and lipopolysaccharide-binding protein) in comparison to baseline. The FMT group had an increase in beneficial gut bacteria, including Ruminococcaceae, and higher levels of short-chain fatty acids like butyrate and isobutyrate, which were linked to the clinical improvements. Additionally, there was a reduction in AUD-related serious adverse events in the FMT treated participants over a six-month period.	Double blind Randomized clinical trial that focused on investigating the efficacy of fecal microbiota transplant for AUD.
S. H. Han et al., 2015	The administration of probiotics (cultured *Lactobacillus subtilis/Streptococcus faecium* 1,500 mg/day) for 7 days resulted in a significant reduction in serum LPS levels, pro-inflammatory cytokines, and improvement in liver enzyme levels compared to the placebo group. The outcome of the study indicated potential therapeutic benefits of probiotics, specifically cultured *Streptococcus faecium/ Lactobacillus subtilis*, in managing alcoholic induced hepatitis.	Randomized-controlled multicenter study that was Double blinded, investigated the effects of probiotics, in the treating of alcohol induced hepatitis.

#### Probiotics, neuroinflammation, and alcohol addiction

Probiotic treatments offer a promising strategy for addressing aspects of alcohol addiction by potentially alleviating symptoms such as anxiety, cravings, dependency, and systemic inflammation (55). They may also contribute to reducing CNS damage and curtailing behavior related to addiction, including excessive alcohol consumption, through their ability to mitigate systemic proinflammatory conditions and neuroinflammation (19, 55).

Alcohol dependency is regarded as an epiphenomenon resulting from systemic neuroinflammation. The precise mechanisms responsible for this association remain incompletely elucidated. Alcohol and its metabolites have been shown to modulate neurotransmitter pathways in the brain, including dopamine circuits, GABA. The impact of alcohol is influenced by the inflammatory response triggered by changes in the gut microbiota composition (56–58). Studies highlight that alcohol-induced dysbiosis can lead to alterations in neurotransmitter release, affecting brain function and mental status (59).

Alcohol significantly impacts GABA neurotransmitter pathways by altering inhibitory neurotransmission and GABAA receptor function. Chronic ethanol exposure leads to a reduction in GABAergic inhibitory postsynaptic currents frequency and changes in expression of GABAA receptor subunit, impairing synaptic inhibition in deep-layer pyramidal neurons of the prefrontal cortex (59). Additionally, ethanol affects the GABAergic system by modulating cAMP-response element binding protein and protein kinase A signaling pathways, with GABA(B) receptors playing a critical role in ethanol’s effects on these pathways (60). Moreover, alcohol affects the production of neuroactive steroids, which quickly change the excitability of neurons, primarily at GABAA receptors. This increases sensitivity to sensitive to ethanol’s behavioral effects and may be a target for therapeutic interventions for alcohol dependency disorders (61).

A complex connection exists between variations in glutamatergic neurotransmission and alcohol dependency. Glutamate, a key excitatory neurotransmitter in the brain, plays a crucial role in the development of alcohol dependency through its modulation of neurochemical, physiological, and behavioral processes (62). Chronic alcohol exposure leads to significant enhancement of glutamatergic activity, particularly through up-regulation of GluN2B-containing N-methyl-D-aspartate receptor, contributing to increased craving for alcohol and maladaptive behaviors (63). The development of alcohol dependency is significantly influenced by alcohol’s activities on both ionotropic and metabotropic glutamate receptors, which change CNS excitability during withdrawal and impact motivational and cognitive behaviors (64). Neuroimaging studies have further elucidated the neurochemical basis of alcohol dependence, emphasizing the role of glutamate alongside GABA and dopamine systems (65). Alcohol interacts directly with GABA receptors, enhancing dopamine release in specific regions such as the ventral tegmental area and the nucleus accumbens (13, 66, 67).

Acute alcohol exposure stimulates dopamine release in the ventral striatum, leading to a euphoric sensation. However, with repeated alcohol use, the brain adapts to the dopamine overload. Alcohol dependence is associated with fewer dopamine D2 receptors in the ventral striatum, contributing to excessive craving for alcohol and an increased risk of relapse (68). Chronic alcohol consumption leads to decreased dopamine transmission in the mesocorticolimbic regions, such as the ventral striatum, which may contribute to anhedonia and decreased reward sensitivity in alcohol-dependent individuals (69).

In addition to affecting the function of the gut and liver, relative abundance and composition gut microbes also have an impact on behavior, mood, and brain function. The bidirectional communication between the brain and gut microbiota and involves immune responses, bacterial metabolites like short-chain fatty acids (SCFAs), and the hypothalamic–pituitary–adrenal axis, all of which can influence neurotransmitter signaling pathways (70). Clinical studies have indicated that increased gut permeability in patients with AUDs is correlated with heightened levels of anxiety, depression, and alcohol craving. Alcohol dependence is associated with intestinal dysbiosis, which leads to increased intestinal permeability and permits the bloodstream to be invaded by bacterial endotoxins like LPS. These endotoxins stimulate inflammatory pathways, which are linked to the psychological symptoms observed in these patients as well as stronger cravings for alcohol (71, 72).

These endotoxins, when present peripherally, can also stimulate the immune system to release proinflammatory cytokines. These cytokines can enter the bloodstream and compromise the blood–brain barrier (BBB), thereby altering neurological processes and escalating a detrimental cycle of inflammation in the brain (73). This not only increases brain dysfunction but also reinforces psychological symptoms and cravings associated with AUDs, further contributing to the progression of the disorder (13).

Individuals diagnosed with hepatic encephalopathy and alcoholism exhibit an elevated ratio of glutamine/glutamate to creatinine. The alteration in the glutamine/glutamate ratio is associated with the pathophysiology of hepatic encephalopathy, where brain levels of glutamine are elevated, thus decreasing the level of glutamate in the brain, which is needed for the synthesis of GABA (74). This reflects the impact of liver dysfunction on neurotransmitter metabolism and brain function in AUD. In ALD, the ammonia produced by gut microbiota is not detoxified, leading to high levels of ammonia in the blood which then reaches the CNS and is taken up by astrocytes. This leads to the early death of astrocytes, causing neurological dysfunction (75).

Probiotics like *Lactobacillus plantarum* and other Lactobacillus and Bifidobacterium species have been shown to produce GABA from glutamate in the gastrointestinal tract. This production of GABA can alleviate symptoms of essential tremor by increasing GABA levels in the cerebellum and reducing neuroinflammation (57, 76, 77). GABA functions as an inhibitory neurotransmitter locally in the gut and also influences the gut-brain axis by modulating the transmission of information from the gut to the central nervous system (57, 77). Additionally, *Lactobacillus plantarum* is linked to the regulation of dopamine levels in the brain, influencing mood and behavior, and regulating glutamate levels in the brain, impacting excitatory neurotransmission (78).

Modulation of the GABA neurotransmission pathway through the use of *Lactobacillus* and *Bifidobacterium* may present a promising therapeutic strategy for addressing AUD and potentially reducing the severity of ALD by influencing GABA levels in the brain (58). Prolonged dietary intervention using a combination of live *Lactobacillus* and *Bifidobacterium* species has been shown to effectively improve cognitive abilities and memory in rats, attributed to the modulation of levels of GABA in the brain (79).

Activation of the AhR in CNS cells plays a crucial role in influencing brain communication through the gut-brain axis (80). Dysbiosis of the gut microbiota, often exacerbated by factors like alcohol abuse, can lead to increased production of metabolites that excessively activate the AhR, potentially exacerbating CNS inflammation. Harmful bacteria can produce pro-inflammatory signals, further exacerbating CNS inflammation through the gut-brain axis (70). The bidirectional gut-brain crosstalk, influenced by AhR activation and gut microbiota metabolites, highlights the intricate relationship between the gut, the brain, and the CNS, and the effects alcohol-induced dysbiosis may have on this axis, offering promising avenues for therapeutic interventions (70, 81). In addition to producing metabolites like indole and tryptamine derivatives and SCFAs, probiotics also help to restore intestinal flora. The AhR may bind to these metabolites or they may function as signaling molecules, activating AhR in a controlled and beneficial manner, thereby reducing CNS inflammation and limiting the severity of AUD (82). Activation of AhR by these beneficial metabolites can promote anti-inflammatory pathways, enhance gut barrier function, and support immune homeostasis, thus reducing inflammation and improving gut-brain communication (83).

Research indicate that the prolonged consumption of dietary supplements containing species of *Lactobacillus* and *Bifidobacterium* can improve memory and cognitive abilities. Studies indicate that probiotics can positively influence cognitive health by modulating the gut-brain axis, leading to improved memory and cognitive performance (84). A study on high alcohol-fed UChB rats treated with probiotic LGG demonstrated an elevation in Fibroblast Growth Factor 21 (FGF21), which serves as a vagal β-Klotho receptor agonist and helps in the reduction of alcohol intake (85). FGF21 decreases dopamine levels in the nucleus accumbens, a key brain region involved in reward and addiction pathways. This reduction in dopamine is mediated by FGF21’s ability to increase the activity and expression activity of the dopamine transporter (86). The heightened absorption of dopamine in the synaptic cleft, influenced by changes in transporter activity induced by the probiotic, plays a crucial role in modulating the rewarding effects and subsequent reduction in alcohol consumption. It is conceivable that the implementation of a probiotics-based supplemental therapy alongside ALD treatment could potentially mitigate the course of the disease by reducing alcohol intake (13, 67).

The influence of probiotics on the modulation of brain receptors in addiction, specifically dopamine receptor 1 and 2 (DR1and DR2), has been the subject of recent investigation. Previous research has documented that the consumption of alcohol and other substances can augment the release of dopamine, eliciting a pleasurable experience and subsequently motivating repetitive behavior area (13, 66, 67).

Gut microbes can synthesize dopamine precursors like phenylalanine and L-dopa, regulating dopamine levels (86). This modulation of dopamine by the microbiome can affect reward circuitry in the brain, where addiction involves dysregulation of dopamine signaling in the mesocorticolimbic pathway, with D1 receptors mediating reinforcement and D2 receptors associated with aversion (69, 87).

A positive correlation was observed between D2R mRNA expression and a reduced presence of bacteria belonging to the *Firmicutes* phylum. This particular phylum includes bacteria from the *Clostridial* order, which, along with the *Ruminococcacea*e and *Lachnospiracea*e families, exhibited a positive correlation with the severity of AUD. Therefore, the restoration of intestinal *Lachnospiraceae* and *Ruminococcaceae* levels through probiotics-based therapy techniques could make D2R a potentially promising target for mitigating AUD severity (88). In contrast to the resilient group of rats, the susceptible group showed a reduced capacity to control alcohol intake, which was related to a noticeable rise in DR1 expression and a decrease in DR2 expression in the striatum. The relationship between variations in the intestinal microbiota and the observed change in susceptible rats suggests a potential role of gut microbiota composition in inhibitory innervations within addiction-related brain circuits (66, 89). The microbiome can influence the brain via the gut-brain axis, with microbial metabolites and inflammatory mediators from a “leaky gut” affecting neurotransmitter systems and brain regions involved in reward and compulsive behaviors (89). Gut microbiota is said to influence the expression of striatal dopamine receptors; however, further research is needed in this regard (67, 88).

Interventions targeting the regulation of gut microbiota in individuals with AUD demonstrated that fecal microbiota obtained from a healthy donor, exhibiting elevated levels of *Lachnospiraceae* and *Ruminococcaceae*, resulted in a temporary decrease in the desire for and intake of alcohol among individuals suffering from alcoholic cirrhosis (72, 88). Reduced levels of *Lachnospiraceae* and *Ruminococcaceae* in patients with AUD have been associated with low levels of intestinal SCFA production. Increased levels of SCFAs in both stool and plasma were noted in individuals with AUD who underwent fecal microbiota transplantation (FMT), positively correlated with the presence of *Lachnospiraceae* and *Ruminococcaceae* bacteria (72, 90). The augmented levels of SCFAs following FMT suggest their potential role in modulating alcohol addiction behavior through their involvement in the gut-brain axis communication (72, 90, 91).

Animals that are fed a diet high in advantageous microorganisms experience less alcohol addiction. Probiotic-treated mice models demonstrated reduced alcohol consumption escalation and relapse following alcohol cessation. Anxiety, depression, and changes in memory were observed in female mice fed alcohol, however, these symptoms were less pronounced in mice with reinforced gut microbiota (92). Probiotic supplementation in AUD patients has been shown to decrease alcohol dependence, alcohol craving and systemic inflammation. By reducing neuroinflammation, probiotics may help relieve alcohol-induced CNS damage and reinforce beneficial effects on addiction (13, 88). Specific probiotic strains like *Lactobacillus rhamnosus* can decrease alcohol intake due to their anti-inflammatory properties (13). Additionally, psychobiotics have been found to influence the gut-brain axis, potentially improving cognitive function and regulating cortisol and interleukin-1β levels in individuals with depression (93). These findings suggest a complex interplay between probiotics, neurotransmitters, and brain receptors, highlighting the promising role. Probiotics may modulate the microbiota-gut-liver-brain axis to treat AUD. By restoring beneficial gut bacteria, probiotics can alleviate alcohol-induced changes and prevent the progression of AUD (9, 13). The intricate relationship between the gut microbiota, neurotransmitter systems, and brain function underscores the potential of probiotics as a therapeutic strategy for mitigating alcohol addiction and its associated pathologies. Table 3 provides a summary of Probiotics and their metabolites and Neuroinflammation.

**Table 3:** Probiotics and their metabolites and Neuroinflammation.

Probiotic/metabolite	Organs targets	Mechanism of action	Type of study	References
*Lactobacillus*	Gut, brain	Produces GABA from glutamate; modulates dopamine and glutamate levels; improves cognitive abilities	Preclinical, clinical	(58, 79, 84)
*Bifidobacterium* species	Gut, Brain	Modulates GABA levels; influences dopamine regulation	Preclinical, clinical	(79, 84)
*Lactobacillus rhamnosus*	Gut, CNS	Reduces alcohol intake; anti-inflammatory properties. Elevates Fibroblast Growth Factor 21and decreases dopamine levels in the nucleus accumbens and helps in the reduction of alcohol intake, reduces oxidative stress.	Animal studies	(85, 86)
*Ruminococcaceae* and *Lachnospiraceae*	Gut, CNS	Decrease in the desire for and intake of alcohol; modulating alcohol addiction behavior; increases D2R mRNA	Preclinical studies, clinical studies	(72, 88, 90)
SCFAs (e.g., Butyrate, Propionate)	Gut, CNS	Modulates neurotransmitter signaling, influences brain function. Enhances gut barrier function, modulates AhR signaling, regulate immune function, maintain gut barrier integrity, reduce systemic inflammation	Preclinical studies	(31, 32, 70, 71, 82, 83)
Tryptophan Metabolites (e.g., Indole, Tryptamine)	Gut, CNS	Act as signaling molecules or ligands for AhR, reduce CNS inflammation, promote anti-inflammatory pathways, enhance gut barrier function, support immune homeostasis. Activates AhR, reduces CNS inflammation. Promotes anti-inflammatory pathways, enhances gut barrier	Preclinical studies	(70, 81, 82)

#### Clinical implications of probiotics in AUD

Probiotics are recognized for their ability to improve health by counteracting pathogenic microbes and boosting the immune system. Probiotics that are most frequently used include *Saccharomyces boulardii, Bifidobacterium*, lactic acid bacteria, and certain Gram-negative bacteria like *Escherichia coli Nissle* 1917. Among these, Lactobacillus and its various strains are particularly recommended for their probiotic properties (9, 41).

Probiotics such as LGG and *Akkermansia muciniphila* are known to boost mucus production, forming a protective gut lining layer and producing antimicrobial substances that suppress pathogenic bacteria and prevent them from adhering to and invading the intestinal epithelium. They produce bacteriocins and other antimicrobial substances that inhibit the growth of pathogenic bacteria, thus protecting the integrity of the gut barrier (44, 94). By reinforcing this barrier, LGG minimizes inflammation-causing LPS translocation from unhealthy gut bacteria into circulation, influences Kupffer cells toward a less inflammatory state, and reduces oxidative stress by decreasing the production of proinflammatory cytokines like TNF-α, IL-6, and IFN-γ, and by increasing levels of enzymes such as SOD and GPX in the liver (45, 95).

LGG is one of the most extensively studied probiotic strains, known for its beneficial effects on both gut barrier function and liver inflammation. Its actions and mechanisms are multifaceted, involving direct interactions with intestinal cells, modulation of the immune system, and effects on the gut-liver axis (45). LGG has demonstrated significant efficacy in mitigating the development of hepatic steatosis (96). The mechanisms by which LGG exerts its protective effects involve the modulation of metabolic pathways and apoptosis regulation within liver cells. LGG restores alcohol-induced reduction in tight junction proteins and prevents alcohol-induced endotoxemia and hepatic steatosis (97). Additionally, the protective effects of LGG are attributed to its ability to reduce hepatic inflammation and fibrosis, ultimately promoting liver health and integrity (96). Research has shown that LGG supplementation can significantly ameliorate alcohol-induced liver injury by reducing hepatic bile acids, enhancing bile acid excretion, and inhibiting hepatic bile acid synthesis, which prevents excessive bile acid-induced liver damage and fibrosis (98).

*A. muciniphila* is a prevalent species in the mammalian gut microbiome, comprising 3 to 5% of the microbial population in the human intestine. The protective effects of *A. muciniphila* on alcohol-induced liver damage have been demonstrated in a mouse model of ALD, where it mitigates ethanol-induced hepatic damage (44). *A. muciniphila* supplementation improved gut barrier function and reduced liver inflammation in a mouse model of ALD. In patients with alcoholic steatohepatitis (ASH) and in mice fed with ethanol, decreased levels of fecal *A. muciniphila* were observed, correlating with the severity of hepatic disease (44, 95). Oral supplementation with *A. muciniphila* reversed this condition, providing protection against ethanol-induced gut leakiness, increased mucus thickness, and improved tight-junction expression (99). By producing more anti-inflammatory cytokines and blocking the NF-κB signaling pathway, which is involved in the expression of proinflammatory genes, *A. muciniphila* has been demonstrated to positively affect the immune system and reduce liver inflammation linked to ALD. *A. muciniphila* also affects bile acid metabolism, which is crucial for liver function and the digestion of fats, and can improve gut barrier function and reduce liver inflammation (45, 95, 99).

A pro-apoptotic member of the Bcl-2 protein family the Bax (Bcl-2-associated X protein) is known to promotes apoptosis. In the context of hepatic steatosis, apoptosis can help eliminate fat-laden hepatocytes, thus preventing the progression of the condition. LGG has been found to regulate the expression of Bax in the liver (42). By modulating the activity of Bax and other apoptotic pathways, LGG can promote the removal of damaged hepatocytes, reducing the burden of hepatic steatosis. This regulation of apoptosis is essential for maintaining liver health and preventing the progression of ALD (100). While apoptosis can be beneficial in removing damaged cells, excessive apoptosis can lead to liver injury. LGG’s ability to regulate apoptosis through Bax suggests that it can strike a balance, promoting cell death where necessary without inducing excessive liver damage (13, 18).

*Bifidobacterium longum* and *Lactobacillus acidophilus* have been shown to influence and inhibit plasma lipid peroxidation. The findings revealed that both strains, when present in the intestine, exhibited varying degrees of effectiveness in safeguarding plasma lipids from oxidation when given at doses of at least 10^8^ CFU/day (101). Additionally, several probiotics can modulate the response of host defense peptides by stimulating the expression of antimicrobial peptides (AMPs). Administration of the probiotic strain *Escherichia coli* Nissle (EcN) and some Lactobacilli species resulted in a significant upregulation of human beta defensin-2 expression within epithelial cells. Administration of other probiotics, such as *Lactobacillus reuteri* at a dose of 4 ng/mL, has been shown to enhance the production of interleukin-22 (IL-22) (39). This cytokine plays a crucial role in the repair and defense of the intestinal mucosa by inducing the expression of AMPs, effectively inhibiting the proliferation of pathogenic bacteria within the intestinal tract in patients with ALD (32, 33). Potential impacts of probiotics on sterol regulatory element-binding proteins (SREBP) activity and lipid metabolism are supported by preclinical studies (102). Probiotics, particularly species such as *Lactobacillus acidophilus*, *Lactobacillus plantarum*, LGG, *Bifidobacterium longum*, and *Bifidobacterium breve*, play a crucial role in safeguarding the intestine and liver from the effects of alcohol. They regulate processes such as hepatic synthesis of fatty acids, catabolism of lipids, lipid transport, and intestinal absorption of lipids (103), mitigate oxidative stress, and reinforce the intestinal epithelial barrier (43). These effects are brought about by probiotics through their anti-inflammatory actions and by modulating bile acid metabolism and signaling pathways that regulate SREBP activity (102, 104). SREBPs serve as transcriptional regulators of lipid biosynthesis in the liver and other tissues (105). They regulate the expression of genes that are involved in the production and uptake of phospholipids, fatty acids, cholesterol, and triglycerides, and their expression is increased in alcohol misuse, leading to the development of hepatic steatosis and elevated levels of plasma triglycerides (102, 105). By altering the gut microbiota composition, probiotics modulate SREBP activity, leading to decreased expression of genes involved in lipid synthesis and contributing to a decreased risk of lipid accumulation in the liver, thus offering a protective mechanism against the development of hepatic steatosis (104, 105). Furthermore, probiotics may contribute to the normalization of SREBP-1c activity, a principal isoform of SREBP involved in fatty acid synthesis. This normalization can lead to a reduction in excessive lipid synthesis and accumulation, partly by enhancing insulin sensitivity (104). Since insulin is a crucial regulator of SREBP-1c, improvements in insulin sensitivity, potentially facilitated by probiotics, could inhibit fatty acid synthesis, offering additional protection against hepatic steatosis (104, 106). SCFAs metabolites produced by probiotics, which reach the liver via portal circulation, have been shown to influence hepatic lipid metabolism, potentially affecting SREBP activity directly in liver cells (103, 107). Probiotic strains like *Lactobacillus*, *Bifidobacterium*, and *Saccharomyces* have shown promise in upregulating PPAR-α expression, promoting fatty acid oxidation, and reducing triglyceride accumulation in the liver. They influence the expression of MTP, improving the liver’s ability to export triglycerides via VLDL, thus reducing lipid accumulation and the risk of fatty liver disease, which is beneficial in mitigating the effects of alcohol consumption (9, 108, 109).

Therapy with probiotics like *Bifidobacterium* (1.0 × 10^8^ CFU) or *Akkermansia* (1.5 × 10^9^ CFU) successfully restores the normal intestinal flora, thereby controlling the integrity of the intestinal barrier (19, 44). In mice given prolonged alcohol treatment, probiotics like *A. muciniphila* and LGG can normalize cytokine levels and enhance the expression of tight junction proteins in the ileum (44, 45). Alcohol-induced upregulation of miR122a leads to a decrease in occludin levels. LGG treatment restores occludin levels to normal by reducing miR122a expression (44).

AMP-activated protein kinase (AMPK), which functions mainly as an energy sensor, is an essential component of cellular energy homeostasis. When activated, AMPK inhibits anabolic processes that use up ATP, like lipogenesis and cholesterol synthesis, and stimulates catabolic processes that produce ATP, like glucose uptake and fatty acid oxidation. Accumulation of fats and cholesterol are two components of hepatic steatosis. LGG and *Lactobacillus plantarum* have been shown to increase the phosphorylation of AMPK in the liver, which enhances the activation of AMPK. LGG helps to restore normal metabolic functions in the liver, protecting against alcohol-induced hepatic steatosis (42, 45). In a clinical trial, individuals who received LGG for 180 days experienced improved liver function and a significant decrease in heavy alcohol consumption when compared to placebo (110).

In a cohort of individuals diagnosed with alcoholic hepatitis, the administration of oral doses of *Lactobacillus subtilis* (1,500 mg/day) and *Streptococcus faecium* (1,500 mg/day) resulted in reduced serum LPS levels, improved serum liver markers, and the restoration of gut microbiota compared to a placebo group (111). Probiotics play a crucial role in safeguarding the intestine and liver from the effects of alcohol by regulating processes such as lipid synthesis, catabolism, and transport. They also mitigate oxidative stress and reinforce the intestinal epithelial barrier (101, 112).

Patients with alcoholic cirrhosis who received treatment with a probiotic regimen that included *Lactobacillus casei* Shirota (6.5 × 10^9^ CFU/65 mL) showed a recovery of neutrophil phagocytic capacity. This probiotic supplementation significantly reduced plasma monocyte chemotactic protein-1, interleukin-1β, interleukin-17a, and macrophage inflammatory protein-1β, all of which promote inflammation and liver injury, compared to the placebo (37). This receptor is involved in the recognition of pathogens and activation of immune responses (52). These findings indicate that probiotic treatment with *Lactobacillus casei* Shirota can improve neutrophil function in patients with alcoholic cirrhosis, potentially by altering IL-10 secretion and TLR4 expression (37, 38).

*Bifidobacterium bifidum* and *Lactobacillus plantarum* 8PA3 have shown promising effects in the treatment of ALD. These probiotics significantly restore normal intestinal flora in individuals with ALD and enhance liver function, as observed through their effect on reducing liver-specific enzymes (116). The administration of prebiotic fructo-oligosaccharides leads to an increase in the population of beneficial bacteria, specifically *Lactobacillus* and *Bifidobacterium*, which have been shown to improve ASH (113). The administration of the prebiotic pectin was found to beneficially affect the restoration of intestinal homeostasis in ALD, evidenced by an increase in the population of goblet cells and the upregulation of defensins Reg3β and Reg3γ. Furthermore, fecal transplantation from mice fed with prebiotic pectin effectively mitigated the occurrence of ALD (115). The combination of prebiotic polysaccharide and *Lactobacillus plantarum* P101 has been shown to regulate the gut microbiota, decrease oxidative stress, and inhibit inflammatory responses by activating Nrf2 and suppressing NFκB in murine models of liver injury (114). In murine models with liver injury treated with *Lactobacillus plantarum*, restoration of gut barrier function has been observed with increased expression of tight junction proteins, reduction in systemic and liver inflammation, and mitigation of damage. These effects are achieved through enhanced mucosal immunity by increased mucin production by goblet cells, elevated production of anti-inflammatory cytokines, and inhibition of the activation of TLR4-mediated MAPK signaling pathway, leading to reduced liver inflammation and injury (117).

Research evaluating the administration of a microstructured synbox loaded with *Lactobacillus plantarum* and epigallocatechin gallate aimed at addressing alcohol-induced liver disease demonstrated a promising synergistic effect in reducing endotoxin levels and hepatic enzyme activity. Furthermore, this synbox system effectively reduced the levels of signaling molecules, including Cyclooxygenase-2, TLR4, CD14, MD2, and key inflammatory biomarkers such as TNF-α, Interleukin 12, and NF-κB (118). VSL#3, a proprietary synergistic combination of eight distinct lactic acid-producing bacterial strains, includes *Lactobacillus acidophilus, Streptococcus thermophilus, Lactobacillus paracasei, Lactobacillus delbrueckii* subsp. *bulgaricus Bifidobacterium breve, Bifidobacterium longum, Bifidobacterium infantis,* and *Lactobacillus plantarum*. These strains are carefully selected for their unique abilities to inhabit various segments of the gastrointestinal tract and work in concert to support gastrointestinal health. VSL#3 is known to enhance the integrity of the gut barrier, modulate immune responses, and foster a balanced gut microbiome. It has been studied for its potential benefits in managing various gastrointestinal conditions like ulcerative colitis, irritable bowel syndrome, and hepatic encephalopathy (38, 118). Introducing the probiotic formula VSL#3 to mice before administering LPS and D-galactosamine effectively prevented the deterioration of colonic barrier integrity. Moreover, this preliminary treatment was associated with diminished bacterial translocation, reduced TNF-α concentrations in tissues, and significantly lessened liver damage. These findings highlight the beneficial effects of VSL#3 in the treatment of liver injury (119, 120).

Probiotics like *LGG, Lactobacillus plantarum, Lactobacillus casei* Shirota*, Lactobacillus acidophilus, Lactobacillus fermentum, Lactobacillus reuteri*, and *Bifidobacterium* species are favored in the treatment of ALD (121). These probiotics have been extensively investigated for their ability to compete with pathogenic bacteria for adhesion sites on the intestinal epithelium, thereby reducing the presence of harmful bacteria. This competitive advantage is attributed to the production of antimicrobial substances such as lactic acid and hydrogen peroxide (121, 122). Additionally, other beneficial flora, such as *Lachnospiraceae*, contribute significantly to the integrity of the intestinal mucosa and produce metabolites like propionate, acetate, and butyrate (91). These SCFAs are generated when these bacteria ferment prebiotics, such as indigestible fibers and fructo-oligosaccharides found in food. SCFAs play crucial roles in maintaining gut health by serving as energy sources for colonocytes (123), regulating immune function, particularly influencing the development and function of regulatory T cells crucial for maintaining immunological tolerance and preventing excessive inflammatory reactions, and inhibiting proinflammatory cytokine production by immune cells (124). They maintain gut barrier integrity, preventing the translocation of pathogenic bacteria and their toxins into the bloodstream, thereby reducing systemic inflammation (123, 124).

The synergistic effects of prebiotics and probiotics support the maintenance of intestinal immunity and integrity. By fostering beneficial microbial growth and suppressing pathogenic species, probiotics and prebiotics alter the gut microbiota composition (123). Prebiotics provide substrates that enhance the growth and activity of probiotics, which, in turn, produce beneficial metabolites like SCFAs. This healthy microbiota competes with pathogenic bacteria for nutrients and attachment sites and stimulates gut-associated lymphoid tissue to support gut health and immune function (123, 125). Table 4 provides a summary of prebiotics and their effects. Table 5 recapitulates the role of various probiotics and their usefulness against ALD.

**Table 4:** Prebiotics and their effects.

Name of the prebiotics/metabolites	Organs targets	Mechanism of action of prebiotics and metabolites	Type of study	References
Prebiotics	Gut	Enhance growth and activity of beneficial gut bacteria, improve gut barrier function, modulate immune response	Clinical trials, animal studies	(7, 9, 41)
Fructo-oligosaccharides (FOS)	Gut	Promote growth of beneficial bacteria like Bifidobacteria, improve gut health, improves alcoholic ASH, enhance mineral absorption	Clinical trials, animal studies, *in vitro*	(38, 52, 53, 113)
Polysaccharides	Gut, immune system	Enhance immune function, improve gut barrier integrity, modulate gut microbiota. Decreased oxidative stress	Clinical trials, animal studies, *in vitro*	(41, 42, 45, 114)
Short-Chain Fatty Acids (SCFAs)	Gut, liver	Produced by fermentation of dietary fibers, enhance gut barrier function, modulate immune response, reduce inflammation	Clinical trials, animal studies, *in vitro*	(17, 22, 31, 32)
Pectin	Gut	Restores intestinal homeostasis in ALD. Increases the population of goblet cells and helps in the upregulation of defensins like the Reg3β and Reg3γ.	Animal studies	(114, 115)

**Table 5:** Probiotics and their mechanisms of action.

Name of the probiotics/metabolite from probiotics	Organs targets	Mechanism of action of probiotics and metabolites	Type of study	References
*Lactobacillus rhamnosus GG (LGG)*	Liver, gut	Enhances gut barrier function, reduces liver inflammation, decreases proinflammatory markers like TNF-α and IL-6, modulates bile acid metabolism, decreases endotoxemia, increases mucin production, reduces oxidative stress, influences Kupffer cells, produces antimicrobial substances. Modulates SREBP activity, influences hepatic lipid metabolism, enhances insulin sensitivity.	Randomized controlled Trials, clinical trials, animal studies, *in vitro*	(13, 18, 19, 42, 52–55)
*Akkermansia muciniphila*	Liver, gut	Increases anti-inflammatory cytokines, inhibits NF-κB signaling, improves gut barrier function, improves tight-junction expression, affects bile acid metabolism, decreases liver inflammation associated with ALD, enhances intestinal mucus layer, reduces gut permeability.	Animal studies, human studies	(9, 11, 24, 41)
*Bifidobacterium longum*	Gut, liver	Reduces oxidative stress, improves gut flora balance, induces antioxidant enzyme activity, upregulates antimicrobial peptides, enhances tight junction protein expression modulates immune response, enhances gut barrier integrity. Inhibits plasma lipid peroxidation, influences hepatic synthesis of fatty acids, regulates bile acid metabolism.	Animal studies, clinical trials	(9, 11, 18, 19, 41, 42, 45)
*Lactobacillus plantarum*	Liver, gut, brain	Produces GABA, modulates dopamine levels, enhances mucosal immunity, reduces systemic inflammation, improves liver function, reduces hepatic steatosis. Increases AMPK phosphorylation, promotes fatty acid oxidation, reduces triglyceride accumulation.	Animal studies, *in vitro*	(9, 11, 18, 19, 42, 45, 53, 55)
*Lactobacillus casei Shirota*	Liver, gut	Normalizes TLR4 expression, modulates gut microbiota, reduces reduces proinflammatory markers and systemic inflammation.	Clinical trials, Animal studies	(11, 18, 19, 41, 42)
*Lactobacillus acidophilus*	Gut	Competes with pathogens, produces antimicrobial substances, enhances gut barrier integrity, modulates immune response, improves digestive health. Inhibits plasma lipid peroxidation, regulates hepatic synthesis of fatty acids, mitigates oxidative stress.	*In vitro*, animal studies	(18, 19, 41, 42, 45)
*Bifidobacterium bifidum*	Gut, liver	Modulates lipid metabolism, enhances liver function, reduces liver-specific enzyme levels, enhances gut microbiota balance, reduces inflammation, enhances intestinal barrier function.	Clinical trials, animal studies, *in vitro*	(11, 18, 19, 41, 42, 45)
*Lactobacillus plantarum* P101	Liver	Regulates gut microbiota, decreases oxidative stress, inhibits inflammatory responses by activating Nrf2, suppressing NFκB	Animal studies	(42, 45, 114)
*Bifidobacterium animalis* subsp. *lactis*	Gut, liver	Demonstrates significant antioxidant activity, reduces oxidative damages, improves gut flora balance, enhances gut barrier function, enhances immune function, reduces gut inflammation, modulates lipid metabolism	*In vitro*, animal studies	(11, 18, 19, 41, 42, 45)
*Lactobacillus reuteri*	Gut, immune system	Enhances IL-22 production, regulates immune response, reduces inflammation, balances gut microbiota, improves gut barrier function, induces expression of AMPs.	Animal studies, *in vitro*	(35, 39, 77, 81)
VSL#3 (Combination of 8 bacterial strains)	Gut, liver	Enhances gut barrier integrity, modulates immune responses, balances gut microbiome, reduces bacterial translocation, diminishes TNF-α concentrations, reduces intestinal inflammation.	Animal studies, clinical trials	(38, 118, 119)
*Escherichia coli* Nissle *(EcN)*	Intestine	Upregulates human beta defensin-2 expression, enhances production of antimicrobial peptides (AMPs)	Preclinical studies	(39, 41)
*Bifidobacterium breve*	Gut, liver	Modulates SREBP activity, influences hepatic lipid metabolism	Preclinical studies	(43, 102–104)
*Saccharomyces boulardii*	Liver	Upregulates PPAR-α expression, reduces triglyceride accumulation, improves lipid transport	Preclinical studies	(41, 108)

## Discussion

The existing body of research has demonstrated encouraging prospects for the use of probiotics to ameliorate the consequences associated with ALD and manage alcohol addiction (9). Probiotics are of significant importance in the regulation of essential pathophysiological processes that are linked to the advancement of liver damage (13). One potential mechanism is the restoration of microbial balance, which serves to counteract dysbiosis caused by prolonged alcohol consumption (32). Alcohol consumption results in a decrease in pathogenic microorganisms and restoration of microbial balance results in a concurrent rise in symbiotic bacteria, thereby augmenting the overall well-being of the gastrointestinal tract (120, 121).

Probiotics effectively reduce the severity of endotoxemia, a medical condition characterized by the presence of bacterial toxins in the bloodstream. Probiotics aid in the mitigation of ALD progression by impeding the transportation of bacterial metabolites to the liver, hence diminishing the inflammatory load on the liver (105). The comprehensive knowledge of the influence of probiotics on ALD necessitates the examination of the gut-microbiota-liver-brain axis (89, 91). Probiotics have been shown to promote digestion, strengthen tight junctions, and safeguard the integrity of intestinal crypts and the mucous layer, leading to a comprehensive enhancement of gastrointestinal well-being. An improved gut environment leads to decreased inflammation, improved barrier function, and less translocation of detrimental chemicals (90, 96).

Probiotics have shown potential in reducing alcohol addiction as well. Research findings indicate that the administration of probiotics has the potential to mitigate many symptoms commonly linked with addiction, including but not limited to feelings of depression, anxiety, alcohol cravings, reliance, and systemic inflammation (90, 92). Probiotics offer a complete strategy for mitigating central nervous system damage and addiction-related behaviors by moderating systemic proinflammatory conditions and neuroinflammation (85, 126, 127). Studies have demonstrated that probiotics can exert significant influence on liver function, particularly in relation to mitigating the detrimental effects of alcohol-induced damage. Notably, strains such as *Lactobacillus reuteri* and LGG have exhibited this capacity (112, 113). Furthermore the impact of probiotics on the immunological response implies a wider range of functions for probiotics in regulating systemic inflammation and pathways associated with the immune system (90).

## Conclusion

The study’s findings demonstrate that probiotics exert a notable influence on both alcohol addiction and alcoholic liver disease. Probiotics have the ability to reinstate equilibrium in the microbiota, mitigate dysbiosis, and foster an environment that counteracts inflammation. This, in turn, diminishes intestinal permeability and averts the movement of bacterial constituents into the systemic circulation. Additionally, they exert regulatory control over the axis connecting the microbiota, gut, liver, and brain, so influencing the functioning of each organ both directly and indirectly. Probiotics have the potential to alleviate systemic proinflammatory conditions and neuroinflammation, both of which play significant roles in the development and progression of alcohol addiction. Certain strains of probiotics, namely *Lactobacillus*, *Bifidobacterium bifidum*, and *Akkermansia*, have been found to improve the integrity of the intestinal barrier in patients diagnosed with AUD. In addition, they possess the ability to control the immune response by reinstating the neutrophil phagocytic capacity and suppressing proinflammatory signals via pathways such as the TLR4 receptor. Probiotics have the ability to regulate neurotransmitter pathways, including those associated with GABA and dopamine, which may have the potential to mitigate alcohol cravings and dependence. Interventions aimed at modulating the gut microbiota, such as the administration FMT, have exhibited potential in the transient reduction of alcohol use and cravings among persons diagnosed with alcoholic cirrhosis. Nevertheless, it is imperative to acknowledge that probiotics ought to be regarded as an integral component of a holistic therapeutic strategy, encompassing behavioral therapies, counseling, and medical supervision.

### Future recommendations

To further elucidate the therapeutic potential of probiotics in treating ALD and AUD, several avenues for future research are recommended. Clinical trials on combined probiotic formulations should be conducted. These trials should aim to evaluate the efficacy of multi-strain probiotic combinations in reducing alcohol induced liver damage, improving gut barrier function, altering gut microbiota composition, and reducing alcohol craving and intake and addiction. Potential strains for these trials include LGG, *Bifidobacterium bifidum*, and *A. muciniphila.* By investigating the synergistic effects of these combinations, we can better understand how multiple probiotic strains may enhance the gut-liver-brain axis and reduce systemic inflammation more effectively than single strains.

Mechanistic studies on probiotics and their metabolites are necessary to identify specific metabolites produced by probiotics and their impacts on gut and liver and CNS functions particularly in relation to AUD. *In vitro* and animal studies should focus on the production of SCFAs, signaling pathways AMP production and production of anti-inflammatory cytokines. *Lactobacillus plantarum* and *Bifidobacterium animalis* subsp. lactis could be promising strains for these studies. Research into the synergistic effects of probiotics and prebiotics should be expanded. Animal studies followed by clinical trials should be conducted to explore how combinations of prebiotics like fructo-oligosaccharides, inulin pectin and other polysaccharides with probiotics such as *Bifidobacterium longum* and *Lactobacillus acidophilus* can enhance gut microbiota composition, SCFA levels, liver function tests, and inflammation markers. Such combinations could enhance the growth and activity of beneficial bacteria more effectively than either alone. There is also a need to assess the impact of probiotics on neuroinflammation and related symptoms in AUD. Preclinical studies followed by clinical trials should investigate how probiotics can particularly modulate GABA and dopamine pathways, how they modulate of other neurotransmitter pathways and reduce neuroinflammation. Such studies could provide insights into how targeting neuroinflammation can reduce the neuropsychiatric symptoms associated with AUD. Additionally, longitudinal cohort studies should be conducted to investigate the long-term effects of probiotic supplementation on alcohol addiction, CNS functions, liver health in patient with AUD. Research should explore how probiotics can modulate the gut-brain axis in the context of alcohol addiction. Behavioral changes, gut microbiota analysis, and brain imaging studies can be used as outcome measures to understand gut-brain interactions and their potential for treating AUD and related disorders. Clinical trials focusing on immune markers are needed to examine how probiotics regulate immune responses in ALD patients. These trials should measure levels of cytokines, immune cell profiles, and incidence of infections, with potential strains including LGG and *Bifidobacterium breve*. Clinical trials comparing the efficacy of different probiotic strains in treating ALD and AUD may be beneficial. Further studies should focus to investigate the role of probiotics in individuals with a genetic predisposition to ALD, such research could reveal how genetic factors influence the response to probiotic treatment in AUD and can provide deeper insights into the therapeutic potential of probiotics in treating ALD and AUD, paving the way for innovative treatments and improved patient outcomes.

## Data Availability

The original contributions presented in the study are included in the article/supplementary material, further inquiries can be directed to the corresponding authors.

## References

[1] Shield K Manthey J Rylett M Probst C Wettlaufer A Parry CDH . National, regional, and global burdens of disease from 2000 to 2016 attributable to alcohol use: a comparative risk assessment study. Lancet Public Health. (2020) 5:e51–61. doi: 10.1016/S2468-2667(19)30231-2, PMID: 31910980

[2] Griswold MG Fullman N Hawley C Arian N Zimsen SRM Tymeson HD . Alcohol use and burden for 195 countries and territories, 1990–2016: a systematic analysis for the global burden of disease study 2016. Lancet. (2018) 392:1015–35. doi: 10.1016/S0140-6736(18)31310-2, PMID: 30146330 PMC6148333

[3] Mathurin P Bataller R. Trends in the management and burden of alcoholic liver disease. J Hepatol. (2015) 62:S38. doi: 10.1016/j.jhep.2015.03.006 25920088 PMC5013530

[4] Koob GF. Neurocircuitry of alcohol addiction: synthesis from animal models. In: Handbook of clinical neurology editors. Sullivan EV Pfefferbaum A. Elsevier (2014). vol 125:33–54. 10.1016/B978-0-444-62619-6.00003-3 25307567

[5] McLellan AT. Substance misuse and substance use disorders: why do they matter in healthcare? Trans Am Clin Climatol Assoc. (2017) 128:112–30. PMID: 28790493 PMC5525418

[6] Guha M. Diagnostic and statistical manual of mental disorders: DSM-5 (5th edition). Reference Reviews (2014) 28:36–7. doi: 10.1108/RR-10-2013-0256,

[7] Kandel D Kandel E. The gateway hypothesis of substance abuse: developmental, biological and societal perspectives. Acta Paediatr. (2015) 104:130–7. doi: 10.1111/apa.12851 25377988

[8] Jonas DE Amick HR Feltner C Bobashev G Thomas K Wines R . Pharmacotherapy for adults with alcohol use disorders in outpatient settings: a systematic review and meta-analysis. JAMA. (2014) 311:1889–900. doi: 10.1001/jama.2014.3628 24825644

[9] Mishra G Singh P Molla M Yimer YS Dinda SC Chandra P . Harnessing the potential of probiotics in the treatment of alcoholic liver disorders. Front Pharmacol. (2023) 14:1212742. doi: 10.3389/fphar.2023.1212742, PMID: 37361234 PMC10287977

[10] Wang S. Historical review: opiate addiction and opioid receptors. Cell Transplant. (2018) 28:233–8. doi: 10.1177/0963689718811060, PMID: 30419763 PMC6425114

[11] Rocco A Compare D Angrisani D Sanduzzi Zamparelli M Nardone G. Alcoholic disease: liver and beyond. World J Gastroenterol. (2014) 20:14652–9. doi: 10.3748/wjg.v20.i40.14652, PMID: 25356028 PMC4209531

[12] Axley PD Richardson CT Singal AK. Epidemiology of alcohol consumption and societal burden of alcoholism and alcoholic liver disease. Clin Liver Dis. (2019) 23:39–50. doi: 10.1016/j.cld.2018.09.011, PMID: 30454831

[13] Fuenzalida C Dufeu MS Poniachik J Roblero JP Valenzuela-Pérez L Beltrán CJ. Probiotics-based treatment as an integral approach for alcohol use disorder in alcoholic liver disease. Front Pharmacol. (2021) 12:729950. doi: 10.3389/fphar.2021.729950 34630107 PMC8497569

[14] Ansari F Pourjafar H Tabrizi A Homayouni A. The effects of probiotics and prebiotics on mental disorders: a review on depression, anxiety, Alzheimer, and autism Spectrum disorders. Curr Pharm Biotechnol. (2020) 21:555–65. doi: 10.2174/1389201021666200107113812, PMID: 31914909

[15] Vrabel M. Preferred reporting items for systematic reviews and meta-analyses. Oncol Nurs Forum. (2015) 42:552–4. doi: 10.1188/15.ONF.552-554 26302284

[16] Sterne JAC Savović J Page MJ Elbers RG Blencowe NS Boutron I . RoB 2: a revised tool for assessing risk of bias in randomised trials. BMJ. (2019) 28:l4898. doi: 10.1136/bmj.l4898 31462531

[17] Catry E Bindels LB Tailleux A Lestavel S Neyrinck AM Goossens JF . Targeting the gut microbiota with inulin-type fructans: preclinical demonstration of a novel approach in the management of endothelial dysfunction. Gut. (2018) 67:271–83. doi: 10.1136/gutjnl-2016-313316 28377388 PMC5868295

[18] Li F Duan K Wang C McClain C Feng W. Probiotics and alcoholic liver disease: treatment and potential mechanisms. Gastroenterol Res Pract. (2016) 2016:5491465. doi: 10.1155/2016/5491465, PMID: 26839540 PMC4709639

[19] Wu W Hu T Liu Y Li P Li Y Sun X . *Bifidobacterium adolescentis* protects against necrotizing enterocolitis and upregulates TOLLIP and SIGIRR in premature neonatal rats. BMC Pediatr. (2017) 17:1. doi: 10.1186/s12887-016-0759-7, PMID: 28056921 PMC5217633

[20] Bernhardt GV Shivappa P Bernhardt K Bhat S Pinto JRT Jhancy M . Markers of inflammation in obese pregnant women: adenosine deaminase and high sensitive C-reactive protein. Eur J Obstet Gynecol Reprod Biol X. (2022) 16:100167. doi: 10.1016/j.eurox.2022.100167, PMID: 36312323 PMC9597103

[21] Bernhardt GV Shivappa P Shantaram M Lokapur V Pinto JRT. Phagocytic and oxidative burst activity of neutrophils in type 2 diabetic patients with foot ulcers. Biomedicine. (2021) 41:776–80. doi: 10.51248/.v41i4.1122

[22] Pathak V Kant R Kumar N. Impact of reactive oxygen species on the progression of human diseases by damaging biomolecules. Biomedicine. (2023) 43:821–4. doi: 10.51248/.v43i3.2439

[23] Mohamed A Hamd E. ROS-mediated inflammatory response in liver damage via regulating the Nrf2/HO-1/NLRP3 pathway in mice with trichloroethylene hypersensitivity syndrome. J Immunotoxicol. (2022) 19:100–8. doi: 10.1080/1547691x.2022.2111003 36070617

[24] Bernhardt GV Pinto JRT Pai VR. Superoxide dismutase: an alternate target for Plasmodium. Biomed Sci. (2009) 20:127–35.

[25] Fan L Zhu Y Cheng J Sun XM Zhang Z Hu H. Low-dose alcohol improves lipid metabolism through store-operated Ca2+ channel-induced PPARγ expression in obese mice. J Food Biochem. (2023) 2023:1–11. doi: 10.1155/2023/2627116

[26] Seitz HK Moreira B Neuman MG. PPathogenesis of Alcoholic Fatty Liver a Narrative Review. Life (Basel). (2023) 13:1662. doi: 10.3390/life13081662 37629519 PMC10455719

[27] Yagai T Nakamura T. Mechanistic insights into the peroxisome proliferator-activated receptor alpha as a transcriptional suppressor. Front Med. (2022) 9:1060244. doi: 10.3389/fmed.2022.1060244 PMC9732035 36507526

[28] Rodriguez Y Dunfield J Roderique T Ni HM. Liver-adipose tissue crosstalk in alcohol-associated liver disease: the role of mTOR. Liver Res. (2022) 6:227–37. doi: 10.1016/j.livres.2022.11.006, PMID: 37124481 PMC10134744

[29] Martínez-Castillo M Altamirano-Mendoza I Sánchez-Valle SS García-Islas LH Hernández-Santillán M Hernández-Barragán A . Immune dysregulation and pathophysiology of alcohol consumption and alcoholic liver disease. Rev Gastroenterol Mex. (2023) 88:136–54. doi: 10.1016/j.rgmxen.2023.03.003, PMID: 36973122

[30] Bishehsari F Magno E Swanson G Desai V Voigt RM Forsyth CB . Alcohol and gut-derived inflammation. Alcohol Res. (2017) 38:163–71. PMID: 28988571 10.35946/arcr.v38.2.02 PMC5513683

[31] Kunst C Schmid S Michalski M Tümen D Buttenschön J Müller M . The influence of gut microbiota on oxidative stress and the immune system. Ther Adv Cardiovasc Dis. (2023) 11:1388–8. doi: 10.3390/biomedicines11051388, PMID: 37239059 PMC10216031

[32] Violi F Nocella C Bartimoccia S Castellani V Carnevale R Pignatelli P . Gut dysbiosis-derived low-grade endotoxemia: a common soil for liver and cardiovascular disease. Kardiol Pol. (2023) 81:563–71. doi: 10.33963/KP.a2023.0115 37191190

[33] Abrehdari Z Pirestani M Allahdini P Safarpour E. Characterization of anti-inflammatory responses of norepinephrine in hepatitis induced by LPS: effects on expression of IL-6, TNF-α and iNOS in liver of mice. Neurochem J. (2014) 8:193–8. doi: 10.1134/S1819712414030027

[34] Hou JJ Ma AH Qin YH. Activation of the aryl hydrocarbon receptor in inflammatory bowel disease: insights from gut microbiota. Front Cell Infect Microbiol. (2023) 13:1279172. doi: 10.3389/fcimb.2023.1279172, PMID: 37942478 PMC10628454

[35] Zhao C Hu X Bao L Wu K Feng L Qiu M . Aryl hydrocarbon receptor activation by *Lactobacillus reuteri* tryptophan metabolism alleviates *Escherichia coli*-induced mastitis in mice. PLoS Pathog. (2021) 17:e1009774. doi: 10.1371/journal.ppat.1009774, PMID: 34297785 PMC8336809

[36] Lee SH Han AR Kim BM Sung MJ Hong SM. *Lactococcus lactis*-fermented spinach juice suppresses LPS-induced expression of adhesion molecules and inflammatory cytokines through the NF-κB pathway in HUVECs. Exp Ther Med. (2022) 23:390. doi: 10.3892/etm.2022.11317, PMID: 35495598 PMC9019603

[37] Macnaughtan J Figorilli F García-López E Lu H Jones H Sawhney R . A double-blind, randomized placebo-controlled trial of probiotic *lactobacillus casei* shirota in stable cirrhotic patients. Nutrients. (2020) 12:1651. doi: 10.3390/nu12061651, PMID: 32498372 PMC7352321

[38] Yousefi B Eslami M Ghasemian A Kokhaei P Salek Farrokhi A Darabi N. Probiotics importance and their immunomodulatory properties. J Cell Physiol. (2019) 234:8008–18. doi: 10.1002/jcp.27559 30317594

[39] Gaudino SJ Beaupre M Lin X Joshi P Rathi S McLaughlin PA . IL-22 receptor signaling in Paneth cells is critical for their maturation, microbiota colonization, Th17-related immune responses, and anti-Salmonella immunity. Mucosal Immunol. (2021) 14:389–401. doi: 10.1038/s41385-020-00348-5, PMID: 33060802 PMC7946635

[40] Patnaude L Mayo M Mario R Wu X Knight H Creamer K . Mechanisms and regulation of IL-22-mediated intestinal epithelial homeostasis and repair. Life Sci. (2021) 271:119195. doi: 10.1016/j.lfs.2021.119195 33581125

[41] Cassard AM Ciocan D. Microbiota, a key player in alcoholic liver disease. Clin Mol Hepatol. (2018) 24:100–7. doi: 10.3350/cmh.2017.0067, PMID: 29268595 PMC6038939

[42] Wang Y Liu L Shen W Jiang Y Sun X Zheng P . *Lactobacillus rhamnosus* GG reduces hepatic TNFα production and inflammation in chronic alcohol-induced liver injury. J Nutr Biochem. (2013) 24:1609–15. doi: 10.1016/j.jnutbio.2013.02.001, PMID: 23618528 PMC3804118

[43] Wang Y Wu Y Wang Y Xu H Mei X Yu D . Antioxidant properties of probiotic bacteria. Nutrients. (2017) 9:521. doi: 10.3390/nu9050521, PMID: 28534820 PMC5452251

[44] Grander C Adolph TE Wieser V Lowe P Wrzosek L Gyongyosi B . Recovery of ethanol-induced *Akkermansia muciniphila* depletion ameliorates alcoholic liver disease. Gut. (2018) 67:891–901. doi: 10.1136/gutjnl-2016-313432, PMID: 28550049

[45] Chen RC Xu LM Du SJ Huang SS Wu H Dong JJ . *Lactobacillus rhamnosus* GG supernatant promotes intestinal barrier function, balances Treg and TH17 cells and ameliorates hepatic injury in a mouse model of chronic-binge alcohol feeding. Toxicol Lett. (2016) 241:103–10. doi: 10.1016/j.toxlet.2015.11.019 26617183

[46] Lee JY Kang CH. Probiotics alleviate oxidative stress in H2O2-exposed hepatocytes and t-BHP-induced C57BL/6 mice. Microorganisms. (2022) 10:234. doi: 10.3390/microorganisms10020234, PMID: 35208690 PMC8877580

[47] Xavier J Duboc H Sokol H. Impaired aryl hydrocarbon receptor ligand production by the gut microbiota is a key factor in metabolic syndrome. Cell Metab. (2018) 28:737–749.e4. doi: 10.1016/j.cmet.2018.07.001, PMID: 30057068

[48] Li Q Zheng T Chen J Li B Zhang Q Yang S . Exploring the benefits of probiotics in gut inflammation and diarrhea—from an antioxidant perspective. Antioxidants. (2023) 12:1342. doi: 10.3390/antiox12071342, PMID: 37507882 PMC10376667

[49] Prem L. Probiotics and human health. Res J Biotechnol. (2023) 18:173–80. doi: 10.25303/1807rjbt1730180

[50] Sumera Z Imtiaz K. Probiotics and their beneficial health effects. Mini Rev Med Chem. (2023) 24:110–25. doi: 10.2174/1389557523666230608163823, PMID: 37291788

[51] Park B Kim SH. Probiotic strains isolated from natural products alleviate gut inflammation in a human epithelial HT-29 in vitro model. Physiology. (2023) 38:38(S1). doi: 10.1152/physiol.2023.38.s1.5766763

[52] Piñero P Singal AK Bataller R Richard K Williams R. Toll-like receptor polymorphisms compromise the inflammatory response against bacterial antigen translocation in cirrhosis. Sci Rep. (2017) 7:46425. doi: 10.1038/srep46425, PMID: 28418003 PMC5394473

[53] Sharma V Garg S Aggarwal S. Probiotics and liver disease. Perm J. (2013) 17:62–7. doi: 10.7812/TPP/12-144, PMID: 24361022 PMC3854811

[54] Shao T Zhao C Li F Gu Z Liu L Zhang L . Intestinal HIF-1α deletion exacerbates alcoholic liver disease by inducing intestinal dysbiosis and barrier dysfunction. J Hepatol. (2018) 69:886–95. doi: 10.1016/j.jhep.2018.05.021, PMID: 29803899 PMC6615474

[55] Timary P Stärkel P Delzenne N Leclercq S. A role for the peripheral immune system in the development of alcohol use disorders? Neuropharmacology. (2017) 122:148–60. doi: 10.1016/j.neuropharm.2017.04.013, PMID: 28400259

[56] Chen G Shi FL Yin W Guo Y Liu A Shuai J . Gut microbiota dysbiosis: the potential mechanisms by which alcohol disrupts gut and brain functions. Front Microbiol. (2022) 13:916765. doi: 10.3389/fmicb.2022.916765, PMID: 35966709 PMC9372561

[57] Gupta H Suk KT Kim DJ. Gut microbiota at the intersection of alcohol, brain, and the liver. J Clin Med. (2021) 10:541. doi: 10.3390/jcm10030541, PMID: 33540624 PMC7867253

[58] Morley KC Lagopoulos J Logge W Baillie A Adams C Haber PS. Brain GABA levels are reduced in alcoholic liver disease: a proton magnetic resonance spectroscopy study. Addict Biol. (2020) 25:e12702. doi: 10.1111/adb.12702, PMID: 30561840

[59] Chivero ET Sil S Kumar M Buch S. Substance use, microbiome and psychiatric disorders. Pharmacol Biochem Behav. (2022) 219:173432. doi: 10.1016/j.pbb.2022.173432 35905802

[60] Dharavath RN Pina-Leblanc C Tang VM Sloan ME Nikolova YS Pangarov P . GABAergic signaling in alcohol use disorder and withdrawal: pathological involvement and therapeutic potential. Front Neural Circuits. (2023) 17:1218737. doi: 10.3389/fncir.2023.1218737, PMID: 37929054 PMC10623140

[61] Benjamin AH John PB Todd KO Melissa AH Leslie M. Chronic ethanol exposure and withdrawal impair synaptic GABAA receptor-mediated neurotransmission in deep-layer prefrontal cortex. Alcohol Clin Exp Res. (2019) 43:822–32. doi: 10.1111/ACER.14015, PMID: 30860602 PMC6502689

[62] Wang Z Zhu X Ni XJ Wen Y Shang D. Knowledge atlas of the involvement of glutamate and GABA in alcohol use disorder: a bibliometric and scientometric analysis. Front Psych. (2022) 13:965142. doi: 10.3389/fpsyt.2022.965142, PMID: 36032235 PMC9411946

[63] Richard LB Hauser SR McClintick JN Rahman S Edenberg HJ Szumlinski KK . Ethanol-associated changes in glutamate reward neurocircuitry: a minireview of clinical and preclinical genetic findings. Prog Mol Biol Transl Sci. (2016) 137:41–85. doi: 10.1016/BS.PMBTS.2015.10.018, PMID: 26809998 PMC4749142

[64] Burnett EJ Chandler LJ Trantham-Davidson H. Glutamatergic plasticity and alcohol dependence-induced alterations in reward, affect and cognition. Prog Neuro-Psychopharmacol Biol Psychiatry. (2016) 65:309–20. doi: 10.1016/J.PNPBP.2015.08.012, PMID: 26341050 PMC4679411

[65] Szumlinski KK Woodward JJ. Chapter 10-Glutamate Signaling in Alcohol Abuse and Dependence. Editor(s): Noronha ABC, Cui C, Harris RA, Crabbe JC. Neurobiol Alcohol Dependence. (2014):173–206. doi: 10.1016/B978-0-12-405941-2.00010-9

[66] Robison J Thakkar KN Diwadkar VA. Cognition and reward circuits in schizophrenia: synergistic. Not Separate Biol Psychiatry. (2020) 87:204–14. doi: 10.1016/j.biopsych.2019.09.021, PMID: 31733788 PMC6946864

[67] Volkow ND Koob GF McLellan AT. Neurobiologic advances from the brain disease model of addiction. N Engl J Med. (2016) 374:363–71. doi: 10.1056/nejmra1511480, PMID: 26816013 PMC6135257

[68] Prasad P Ambekar A Vaswani M. Dopamine D2 receptor polymorphisms and susceptibility to alcohol dependence in Indian males: a preliminary study. BMC Med Genet. (2010) 11:24. doi: 10.1186/1471-2350-11-24, PMID: 20146828 PMC2829542

[69] Jayaram-Lindström N Ericson M Steensland P Jerlhag E. Dopamine and alcohol dependence: from bench to clinic. Recent advances in drug addiction research and clinical applications. INTECH. (2016). doi: 10.5772/63144

[70] Magni G Riboldi B Ceruti S. Modulation of glial cell functions by the gut–brain axis: a role in neurodegenerative disorders and pain transmission. Cells. (2023) 12:1612. doi: 10.3390/cells12121612, PMID: 37371082 PMC10297219

[71] Wolstenholme JT Duong NK Brocato ER Bajaj JS. Gut-liver-brain axis and alcohol use disorder: treatment potential of fecal microbiota transplantation. Alcohol Res. (2024) 44:01. doi: 10.35946/arcr.v44.1.01 38322428 PMC10843328

[72] Bajaj JS Gavis EA Fagan A Kassam ZA Sikaroodi M Gillevet PM . A randomized clinical trial of fecal microbiota transplant for alcohol use disorder. Hepatology. (2021) 73:1688–700. doi: 10.1002/hep.31496, PMID: 32750174

[73] Caspani G Swann JR. Small talk: microbial metabolites involved in the signaling from microbiota to brain. Curr Opin Pharmacol. (2019) 48:99–106. doi: 10.1016/j.coph.2019.08.001, PMID: 31525562

[74] Kakulavarapu VR Jayakumar AR Norenberg MD. Glutamine in the pathogenesis of acute hepatic encephalopathy. Neurochem Int. (2012) 61:575–80. doi: 10.1016/j.neuint.2012.01.012 22285152

[75] Görg B Karababa A Häussinger D. Hepatic encephalopathy and astrocyte senescence. J Clin Exp Hepatol. (2018) 8:294–300. doi: 10.1016/j.jceh.2018.05.003, PMID: 30302047 PMC6175776

[76] Zhong HJ Wang SQ Zhang RX Zhuang YP Li L Yi SZ . Supplementation with high-GABA-producing *Lactobacillus plantarum* L5 ameliorates essential tremor triggered by decreased gut bacteria-derived GABA. Transl Neurodegener. (2023) 12:58. doi: 10.1186/s40035-023-00391-9, PMID: 38093327 PMC10717605

[77] Strandwitz P. Neurotransmitter modulation by the gut microbiota. Brain Res. (2018) 1693:128–33. doi: 10.1016/j.brainres.2018.03.015, PMID: 29903615 PMC6005194

[78] Diez-Gutiérrez L San Vicente L Barrón LJR Villarán MDC Chávarri M. Gamma-aminobutyric acid and probiotics: multiple health benefits and their future in the global functional food and nutraceuticals market. J Funct Foods. (2020) 64:103669. doi: 10.1016/j.jff.2019.103669

[79] O’Hagan C Li JV Marchesi JR Plummer S Garaiova I Good MA. Long-term multi-species Lactobacillus and Bifidobacterium dietary supplement enhances memory and changes regional brain metabolites in middle-aged rats. Neurobiol Learn Mem. (2017) 144:36–47. doi: 10.1016/j.nlm.2017.05.015, PMID: 28602659

[80] Bicknell B Liebert A Borody TJ Herkes GK McLachlan CS Kiat H. Neurodegenerative and neurodevelopmental diseases and the gut-brain axis: the potential of therapeutic targeting of the microbiome. Int J Mol Sci. (2023) 24:9577. doi: 10.3390/ijms24119577, PMID: 37298527 PMC10253993

[81] Munshi R Solanki S Karande AA Ranganathan P. Emerging role of gut microbiota dysbiosis in neuroinflammation and neurodegeneration. Front Neurol. (2023) 14:1149618. doi: 10.3389/fneur.2023.1149618, PMID: 37255721 PMC10225576

[82] Hubbard TD Murray IA Perdew GH. Indole and tryptophan metabolism: endogenous and dietary routes to ah receptor activation. Drug Metab Dispos. (2015) 43:1522–35. doi: 10.1124/dmd.115.064246, PMID: 26041783 PMC4576673

[83] Rothhammer V Mascanfroni ID Bunse L Takenaka MC Kenison JE Mayo L . Type I interferons and microbial metabolites of tryptophan modulate astrocyte activity and central nervous system inflammation via the aryl hydrocarbon receptor. Nat Med. (2016) 22:586–97. doi: 10.1038/nm.4106, PMID: 27158906 PMC4899206

[84] Fekete M Lehoczki A Major D Fazekas-Pongor V Csípő T Tarantini S . Exploring the influence of gut–brain axis modulation on cognitive health: a comprehensive review of prebiotics, probiotics, and symbiotics. Nutrients. (2024) 16:789. doi: 10.3390/nu16060789, PMID: 38542700 PMC10975805

[85] Ezquer F Uribe P Bahamondes J Morales P Hernandez L Allende F . Innate gut microbiota predisposes to high alcohol consumption. Addict Biol. (2021) 26:e13018–4. doi: 10.1111/adb.13018, PMID: 33508889

[86] Lewin-Epstein O Jaques Y Feldman MW Kaufer D Hadany L. Evolutionary modeling suggests that addictions may be driven by competition-induced microbiome dysbiosis. Commun Biol. (2023) 6:782. doi: 10.1038/s42003-023-05099-0 37495841 PMC10372008

[87] García-Cabrerizo R Carbia C O’Riordan KJ Schellekens H Cryan JF. Microbiota-gut-brain axis as a regulator of reward processes. J Neurochem. (2021) 157:1495–524. doi: 10.1111/jnc.15284, PMID: 33368280

[88] Jadhav KS Davison D Jagannathan L Ghate M Selvaraj S Lee JS . Gut microbiome correlates with altered striatal dopamine receptor expression in a model of compulsive alcohol seeking. Neuropharmacology. (2018) 141:249–59. doi: 10.1016/j.neuropharm.2018.08.026 30172845

[89] Xuan F Ti C Jingda C Bo L Yaohui Z Xiaojie Z. The microbiome–gut–brain axis, a potential therapeutic target for substance-related disorders. Front Microbiol. (2021) 12:738401. doi: 10.3389/fmicb.2021.738401 34690981 PMC8526971

[90] Biazzo M Deidda G. Fecal microbiota transplantation as new therapeutic avenue for human diseases. J Clin Med. (2022) 11:4119. doi: 10.3390/jcm11144119, PMID: 35887883 PMC9320118

[91] Yue X Wen S Long-Kun D Man Y Chang S Min Z . Three important short-chain fatty acids (SCFAs) attenuate the inflammatory response induced by 5-FU and maintain the integrity of intestinal mucosal tight junction. BMC Immunol. (2022) 23:19. doi: 10.1186/s12865-022-00495-3, PMID: 35448938 PMC9027456

[92] Pizarro N Burdeos GP Guarner F Molero Y Gual A Castells X . Sex-specific effects of synbiotic exposure in mice on addictive-like behavioral alterations induced by chronic alcohol intake are associated with changes in specific gut bacterial taxa and brain tryptophan metabolism. Front Nutr. (2021) 8:750333. doi: 10.3389/fnut.2021.750333, PMID: 34901109 PMC8662823

[93] Skowron K Budzyńska A Wiktorczyk-Kapischke N Chomacka K Grudlewska-Buda K Wilk M . The role of psychobiotics in supporting the treatment of disturbances in the functioning of the nervous system—a systematic review. Int J Mol Sci. (2022) 23:7820. doi: 10.3390/ijms23147820, PMID: 35887166 PMC9319704

[94] Gu Y Qin X Zhou G Wang C Mu C Liu X . *Lactobacillus rhamnosus* GG supernatant promotes intestinal mucin production through regulating 5-HT4R and gut microbiota. Food Funct. (2022) 13:12144–55. doi: 10.1039/d2fo01900k, PMID: 36326009

[95] Sparfel L Ratodiarivony S Boutet-Robinet E Ellero-Simatos S Jolivet-Gougeon A. Akkermansia muciniphila and alcohol-related liver diseases. A systematic review. Mol Nutr Food Res. (2024) 68:e2300510. doi: 10.1002/mnfr.202300510 38059838

[96] Jang HR Park HJ Kang D Chung H Nam MH Lee HJ . A protective mechanism of probiotic Lactobacillus against hepatic steatosis via reducing host intestinal fatty acid absorption. Exp Mol Med. (2019) 51:1–14. doi: 10.1038/s12276-019-0293-4 PMC6802638 31409765

[97] Ge Y Sun H Xu L Zhang W Lv J Chen Y. The amelioration of alcohol-induced liver and intestinal barrier injury by *Lactobacillus rhamnosus* Gorbach-Goldin (LGG) is dependent on interleukin 22 (IL-22) expression. Bioengineered. (2022) 13:12650–60. doi: 10.1080/21655979.2022.2070998, PMID: 35603884 PMC9275995

[98] Liu Y Chen K Li F Gu Z Liu Q He L . Probiotic *Lactobacillus rhamnosus* GG prevents liver fibrosis through inhibiting hepatic bile acid synthesis and enhancing bile acid excretion in mice. Hepatology. (2020) 71:2050–66. doi: 10.1002/hep.30975, PMID: 31571251 PMC7317518

[99] Grander C Grabherr F Spadoni I Enrich B Oberhuber G Rescigno M . The role of gut vascular barrier in experimental alcoholic liver disease and *A. muciniphila* supplementation. Gut Microbes. (2020) 12:1851986. doi: 10.1080/19490976.2020.1851986, PMID: 33382359 PMC7714498

[100] Behzadi E Hosseini HM Fooladi AA. The inhibitory impacts of *Lactobacillus rhamnosus* GG-derived extracellular vesicles on the growth of hepatic cancer cells. Microb Pathog. (2017) 110:1–6. doi: 10.1016/j.micpath.2017.06.016, PMID: 28634130

[101] Amaretti A Di Nunzio M Pompei A Raimondi S Rossi M Bordoni A. Antioxidant properties of potentially probiotic bacteria: in vitro and in vivo activities. Appl Microbiol Biotechnol. (2013) 97:809–17. doi: 10.1007/s00253-012-4241-7 22790540

[102] Alves CC Waitzberg DL de Andrade LS Dos Santos AL Reis MB Guanabara CC . Prebiotic and synbiotic modifications of beta oxidation and lipogenic gene expression after experimental hypercholesterolemia in rat liver. Front Microbiol. (2017) 8:2010. doi: 10.3389/fmicb.2017.02010, PMID: 29089934 PMC5650986

[103] Kumar M Nagpal R Kumar R Hemalatha R Verma V Kumar A . Cholesterol-lowering probiotics as potential biotherapeutics for metabolic diseases. Exp Diabetes Res. (2012) 2012:902917. doi: 10.1155/2012/902917, PMID: 22611376 PMC3352670

[104] Wei Q Zhang S Chu W Wang X Zhou F Han L . JAZF1 ameliorates age and diet-associated hepatic steatosis through SREBP-1c-dependent mechanism. Cell Death Dis. (2018) 9:859. doi: 10.1038/s41419-018-0923-0, PMID: 30154417 PMC6113258

[105] Duan Y Gong K Xu S Zhang F Meng X Han J. Regulation of cholesterol homeostasis in health and diseases: from mechanisms to targeted therapeutics. Signal Transduct Target Ther. (2022) 7:265. doi: 10.1038/s41392-022-01125-5, PMID: 35918332 PMC9344793

[106] Ge JL Anglade D Berk PD. Regulation of hepatocellular fatty acid uptake in mouse models of fatty liver disease with and without functional leptin signaling: roles of NfKB and SREBP-1C and the effects of spexin. Semin Liver Dis. (2016) 36:360–72. doi: 10.1055/s-0036-1597248 27997977

[107] Xiong RG Zhou DD Wu SX Huang SY Saimaiti A Yang ZJ . Health benefits and side effects of short-chain fatty acids. Food Secur. (2022) 11:2863. doi: 10.3390/foods11182863, PMID: 36140990 PMC9498509

[108] Lopez-Escalera S Lund ML Hermes GDA Choi BS Sakamoto K Wellejus A. In vitro screening for probiotic properties of Lactobacillus and Bifidobacterium strains in assays relevant for non-alcoholic fatty liver disease prevention. Nutrients. (2023) 15:2361. doi: 10.3390/nu15102361, PMID: 37242245 PMC10224198

[109] Puttarat N Kasorn A Vitheejongjaroen P Chantarangkul C Tangwattanachuleeporn M Taweechotipatr M. Beneficial Effects of Indigenous Probiotics in High-Cholesterol Diet-Induced Hypercholesterolemic Rats. Nutrients. (2023) 15:2710. doi: 10.3390/nu15122710 37375614 PMC10301077

[110] Vatsalya MD Feng W Kong M Hu H Szabo G McCullough A . The beneficial effects of Lactobacillus GG therapy on liver and drinking assessments in patients with moderate alcohol-associated hepatitis. Am J Gastroenterol. (2023) 118:1457–60. doi: 10.14309/ajg.0000000000002283, PMID: 37040544 PMC10524173

[111] Han SH Suk KT Kim DJ Kim MY Baik SK Kim YD . Effects of probiotics (cultured *Lactobacillus subtilis/Streptococcus faecium)*) in the treatment of alcoholic hepatitis: randomized controlled multicenter study. Eur J Gastroenterol Hepatol. (2015) 27:1300–6. doi: 10.1097/MEG.0000000000000458, PMID: 26302024

[112] Yan AW Schnabl B. Bacterial translocation and changes in the intestinal microbiome associated with alcoholic liver disease. World J Hepatol. (2012) 4:110–8. doi: 10.4254/wjh.v4.i4.110 22567183 PMC3345535

[113] Mijangos-Trejo A Nuño-Lambarri N Barbero-Becerra V Uribe-Esquivel M Vidal-Cevallos P Chávez-Tapia N. Prebiotics and probiotics: therapeutic tools for nonalcoholic fatty liver disease. Int J Mol Sci. (2023) 24:14918. doi: 10.3390/ijms241914918, PMID: 37834367 PMC10573697

[114] Xu X Liu S Zhao Y Wang M Hu LT Li WJ . Combination of *Houttuynia cordata* polysaccharide and Lactiplantibacillus plantarum P101 alleviates acute liver injury by regulating gut microbiota in mice. J Sci Food Agric. (2022) 102:6848–57. doi: 10.1002/jsfa.12046, PMID: 35639719

[115] Wang Q Li Y Sun Y Tian J Chen J Li X . Identification of a protective Bacteroides strain of alcoholic liver disease and its synergistic effect with pectin. Appl Microbiol Biotechnol. (2022) 106:3735–49. doi: 10.1007/s00253-022-11946-7, PMID: 35554627

[116] Sung H Kim SW Hong M Suk KT. Microbiota-based treatments in alcoholic liver disease. World J Gastroenterol. (2016) 22:6673–82. doi: 10.3748/wjg.v22.i29.6673, PMID: 27547010 PMC4970471

[117] Li H Cheng S Huo J Dong K Ding Y Man C . *Lactobacillus plantarum* J26 alleviating alcohol-induced liver inflammation by maintaining the intestinal barrier and regulating MAPK signaling pathways. Nutrients. (2022) 15:190. doi: 10.3390/nu15010190, PMID: 36615846 PMC9824527

[118] Rishi P Arora S Kaur UJ Chopra K Kaur IP. Better management of alcohol liver disease using a 'Microstructured Synbox' system comprising *L. plantarum* and EGCG. PLoS One. (2017) 12:e0168459. doi: 10.1371/journal.pone.0168459, PMID: 28060832 PMC5217831

[119] Li J Hollenhorst MI. Probiotic mixture VSL#3 prevents ulcerative colitis-associated carcinogenesis in mice and cells by regulating the inflammatory and Wnt/β-catenin pathway. Chin Med J. (2022) 135:2357–9. doi: 10.1097/cm9.0000000000002035, PMID: 35672115 PMC9771233

[120] Yan F Polk DB. Characterization of a probiotic-derived soluble protein which reveals a mechanism of preventive and treatment effects of probiotics on intestinal inflammatory diseases. Gut Microbes. (2012) 3:25–8. doi: 10.4161/gmic.19245, PMID: 22356855 PMC3337122

[121] Agazzi A Ferroni M Fanelli A . Effects of species-specific probiotic addition to milk replacer on calf health and performance during the first month of life. Ann Anim Sci. (2014) 14:101–15. doi: 10.2478/aoas-2013-0089

[122] Sagheddu V Uggeri F Belogi L Remollino L Brun P Bernabè G . The biotherapeutic potential of *Lactobacillus reuteri* characterized using a target-specific selection process. Front Microbiol. (2020) 11:532. doi: 10.3389/fmicb.2020.00532, PMID: 32351460 PMC7176361

[123] Markowiak P Ślizewska K. Effects of probiotics, prebiotics, and synbiotics on human health. Nutrients. (2017) 9:1021. doi: 10.3390/nu9091021, PMID: 28914794 PMC5622781

[124] Liu XF Shao JH Liao YT Wang LN Jia Y Dong PJ . Regulation of short-chain fatty acids in the immune system. Front Immunol. (2023) 14:1186892. doi: 10.3389/fimmu.2023.1186892, PMID: 37215145 PMC10196242

[125] Xiao X Nakatsu G Jin Y Wong S Yu J Lau JYW. Gut microbiota mediates protection against enteropathy induced by indomethacin. Sci Rep. (2017) 7:40317. doi: 10.1038/srep40317, PMID: 28067296 PMC5220306

[126] Tiwari V Veeraiah P Subramaniam V Patel A. Differential effects of ethanol on regional glutamatergic and GABAergic neurotransmitter pathways in mouse brain. J Neurochem. (2014) 128:628–40. doi: 10.1111/jnc.12508, PMID: 24164397

[127] Xu J Wu F Li Y Wang F Lin W Qian S . Fibroblast growth factor 21 associating with serotonin and dopamine in the cerebrospinal fluid predicts impulsivity in healthy subjects. BMC Neurosci. (2021) 22:68. doi: 10.1186/s12868-021-00676-7, PMID: 34800969 PMC8605581

